# Computational Molecular Docking and Simulation-Based Assessment of Anti-Inflammatory Properties of *Nyctanthes arbor-tristis Linn* Phytochemicals

**DOI:** 10.3390/ph17010018

**Published:** 2023-12-22

**Authors:** Varish Ahmad, Mohammad Imran Khan, Qazi Mohammad Sajid Jamal, Faisal A. Alzahrani, Raed Albiheyri

**Affiliations:** 1 Health Information Technology Department, The Applied College, King Abdulaziz University, Jeddah 21589, Saudi Arabia; vaahmad@kau.edu.sa; 2 Centre for Artificial Intelligence in Precision Medicines, King Abdulaziz University, Jeddah 21589, Saudi Arabia; 3 Research Centre, King Faisal Specialist Hospital and Research Centre, P.O. Box 40047, Jeddah 21499, Saudi Arabia; 4 Department of Health Informatics, College of Public Health and Health Informatics, Qassim University, Al Bukayriyah 52741, Saudi Arabia; 5 Embryonic Stem Cell Unit, Department of Biochemistry, Faculty of Science, King Fahad Center for Medical Research, King Abdulaziz University, Jeddah 21589, Saudi Arabia; faahalzahrani@kau.edu.sa; 6 Department of Biological Sciences, Faculty of Science, King Abdulaziz University, Jeddah 21589, Saudi Arabia; ralbiheyri@kau.edu.sa; 7 Centre of Excellence in Bionanoscience Research, King Abdulaziz University, Jeddah 21589, Saudi Arabia

**Keywords:** *Nyctanthes arbor-tristis Linn*, natural products, anti-inflammatory activity, cyclooxygenase enzyme inhibition, binding affinity, molecular docking simulation

## Abstract

The leaves, flowers, seeds, and bark of the *Nyctanthes arbor-tristis Linn* plant have been pharmacologically evaluated to signify the medicinal importance traditionally described for various ailments. We evaluated the anti-inflammatory potentials of 26 natural compounds using AutoDock 4.2 and Molecular Dynamics (MDS) performed with the GROMACS tool. SwissADME evaluated ADME (adsorption, distribution, metabolism, and excretion) parameters. Arb_E and Beta-sito, natural compounds of the plant, showed significant levels of binding affinity against COX-1, COX-2, PDE4, PDE7, IL-17A, IL-17D, TNF-α, IL-1β, prostaglandin E2, and prostaglandin F synthase. The control drug celecoxib exhibited a binding energy of −9.29 kcal/mol, and among the tested compounds, Arb_E was the most significant (docking energy: −10.26 kcal/mol). Beta_sito was also observed with high and considerable docking energy of −8.86 kcal/mol with the COX-2 receptor. COX-2 simulation in the presence of Arb_E and control drug celecoxib, RMSD ranged from 0.15 to 0.25 nm, showing stability until the end of the simulation. Also, MM-PBSA analysis showed that Arb_E bound to COX-2 exhibited the lowest binding energy of −277.602 kJ/mol. Arb_E and Beta_sito showed interesting ADME physico-chemical and drug-like characteristics with significant drug-like effects. Therefore, the studied natural compounds could be potential anti-inflammatory molecules and need further in vitro/in vivo experimentation to develop novel anti-inflammatory drugs.

## 1. Introduction

*Nyctanthes arbor-tristis Linn* (NAT) is a medicinal plant of Indian origin that belongs to the family Oleaceae. It is also used as an ornamental plant and is commonly known as night jasmine or harsingar in India. It has a wide geographic distribution in the sub-Himalayan areas and south to the Godavari. The plant has a height of nearly 10 m. The leaves are hairy, decussately rough, opposite, and simple. The flowers are arranged at the ends of the branches. The plant is well cultivated in loamy soil with a sufficient supply of water, and it demands environments ranging from full sunshine to partial shade. Flowering typically takes place from July to October [1,2].

The leaves, flowers, seeds, and bark of this plant have been pharmacologically evaluated to verify the medicinal importance that has traditionally been described for various ailments, such as fever, rheumatism, sciatica, arthritis, malaria, and skin diseases [2,3].

Phytochemicals like glycoside, flavonoids, oleanic acid, tannic acid, essential oils, carotene, lupeol, friedeline, benzoic acid, and glucose have been reported for their therapeutic significance. Among the various pharmacological activities of NAT phytochemicals, their anti-inflammatory potential has attracted considerable attention in recent years. Inflammation is a complicated biological response to adverse stimuli like pathogens, damaged cells, and irritants, as well as their toxins. Immune cells are sensitized in this process and release pro-inflammatory mediators, such as prostaglandins, cytokines, and chemokines. Numerous illnesses, including rheumatoid arthritis, diabetes, atherosclerosis, and cancer, are linked to chronic inflammation [4].

Therefore, finding natural anti-inflammatory agents that can modulate the inflammatory response without causing adverse effects is significant. Researchers have noted that NAT has anti-inflammatory properties, and this has been proven with many animal models and cell lines [5,6]. There have been reports of strong medicinal effects from the plant’s leaves. Phytochemically, the leaves of the plant have been described to contain a class of polyphenolic compounds (flavonoids), such as kaempferol, quercetin, rutin, and astragalin that have antioxidant, anti-inflammatory, and antimicrobial properties. A class of lipophilic compounds (triterpenoids) that has been reported in the leaves of plants includes ursolic acid, β-sitosterol, oleanolic acid, and lupeol, and these compounds are claimed to have anti-inflammatory, anticancer, and antidiabetic effects [7]. The identified phytochemicals of this plant available in the PubChem database are oleanolic acid, friedelin, 6beta-hydroxyloganin, arborside A, arborside B, 6-beta-hydroxy-loganin, calceolarioside A, astragalin, sitogluside, methyl (1S,4aS)-6-hydroxy-5-[(E)-3-(4-methoxyphenyl)prop-2-enoyl]oxy-7-methyl-1-[(2S,3R,4S,5S,6R)-3,4,5-trihydroxy-6-(hydroxymethyl)oxan-2-yl]oxy-1,4a,5,6,7,7a-hexahydrocyclopenta[c]pyran-4-carboxylate, arbortristoside B, nyctanthic acid, arbortristoside E, arbortristoside D, arbortristoside C, 7-O-(3,4-dihydrocycinnamoyl)nyctanthoside, arborside D, etc.

Nyctanthine and arbortristosides are alkaloids that are members of a class of nitrogen-containing compounds that have been reported to have diverse biological activities, such as analgesic, antispasmodic, and antimalarial effects. The phenolic acids, lignans, coumarins, fatty acids, and essential oils have also been reported to have various pharmacological effects, such as antioxidant, antibacterial, antifungal, and antiviral properties [8].

The hydrophilic portion of the alcoholic extract of NAT leaves was reported to significantly suppress cotton pellet granuloma and paw edema in rats induced by carrageenan [9]. LPS-induced NO generation and iNOS expression in RAW 264.7 macrophages were inhibited by the methanol-based extract of NAT leaves, demonstrating significant anti-inflammatory effects [8]. The ethanolic extract of NAT leaves reduced the levels of pro-inflammatory cytokines, such as tumor necrosis factor-alpha (TNF-α), interleukin-1 beta (IL-1β), and interleukin-6 (IL-6), in LPS-stimulated mouse peritoneal macrophages. Moreover, some bioactive compounds isolated from NAT leaves have been identified as potential inhibitors of Janus kinases (JAKs), which are key enzymes involved in the signal transduction of cytokines and play a significant role in the pathogenesis of rheumatoid arthritis [5,10]. These studies suggest that NAT leaves possess an effective anti-inflammatory potential that may be significant in curing inflammatory disorders. However, further studies are needed to elucidate the exact mechanisms of action and the safety profiles of NAT leaves and their constituents. In this study, we aimed to conduct a computational study that verified the anti-inflammatory activity of NAT phytochemicals and highlighted the future pharmaceutical potential of this plant for the development of non-significant anti-inflammatory agents. Some of the phytochemicals that were isolated from NAT leaves and tested for their anti-inflammatory activities in lipopolysaccharide-stimulated RAW 264.7 macrophages included astragalin, an inhibitor of nitric oxide, and inducible nitric oxide synthase (a flavonoid glycoside) [11].

β-sitosterol triterpene decreased the production of IL-6, TNF, and IL-1 in LPS-stimulated mice peritoneal macrophages. It has been reported that leaves of NAT are rich in polyphenolic compounds (flavonoids) like kaempferol, quercetin, astragalin, and rutin [4,12,13]. These phytochemicals of the flavonoid class have been reported to have antioxidant, anti-inflammatory, and antimicrobial properties [14].

A group of nitrogen-containing alkaloids exhibit various biological behaviors, including analgesic, antispasmodic, and antimalarial properties. Arbortristosides A and B are two alkaloids that were reported to inhibit the activity of JAKs and reduce the levels of IL-1β, IL-6, and TNF-α in LPS-stimulated macrophages [15]. Triterpenoids, a class of lipophilic phytochemicals such as beta-sitosterol, ursolic acid, oleanolic acid, and lupeol [12], have been reported to have anticancer, antidiabetic, and anti-inflammatory effects [14]. 

The effectiveness of these phytochemicals compared to synthetic anti-inflammatory drugs may vary depending on the type and severity of the inflammation, the dose and route of administration, the bioavailability and metabolism of the compounds, and the possible interactions with other drugs or substances. However, some studies have reported that these phytochemicals have anti-inflammatory activities comparable or superior to some commonly used synthetic drugs, such as indomethacin, diclofenac, ibuprofen, and prednisolone [16,17,18,19].

For example, astragalin has been described to have a similar inhibitory effect on NO production as indomethacin in LPS-stimulated RAW 264.7 macrophages. At the same time, β-sitosterol was reported to have a more substantial inhibitory effect on IL-6 production than diclofenac in LPS-stimulated mouse peritoneal macrophages [18]. Moreover, arbortristosides A and B have been observed to have a more potent inhibitory effect on JAKs than prednisolone in LPS-stimulated RAW 264.7 macrophages. However, more clinical trials are needed to confirm the safety and efficacy of these phytochemicals in human subjects with inflammatory diseases [17].

**Figure 1:**
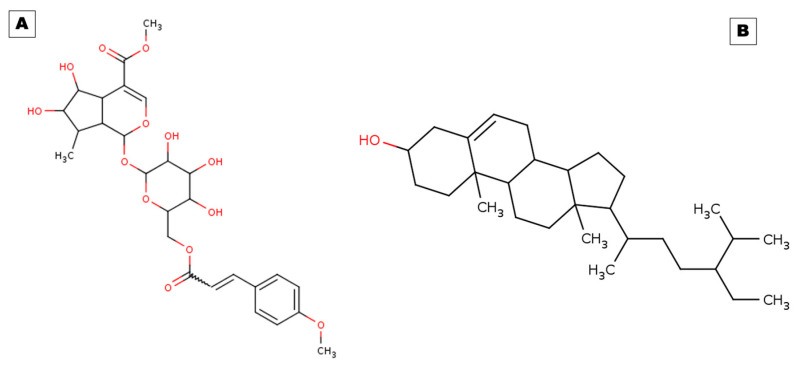
Two-dimensional structural representations of (**A**) arbortristoside E and (**B**) beta-sitosterol.

Combating persistent inflammation and raising patients’ standards of healthy life necessitate the discovery of novel anti-inflammatory medications. Scientists are using natural resources, including plants, to create new medicines with fewer adverse effects. This study aimed to identify potential natural anti-inflammatory molecules interacting with different inflammatory mediators or receptors to potentiate therapeutic effects. This is significant for accelerating their use in therapy at low doses. In this regard, we conducted computational studies based on screening, molecular docking, and simulations of the phytochemicals arbortristoside E and beta-sitosterol of this plant. Arbortristoside E (PubChem ID:14632884) has a molecular weight of 566.5 g/mol, and its molecular formula is C_27_H_34_O_13_. Esters, hydroxyls, and aromatic rings are just a few of the functional groups that make up its complicated structure. Beta-sitosterol (PubChem ID:222284) belongs to the class of phytosterols with beta-hydroxy groups. It has a molecular weight of 414.7 g/mol, and its molecular formula is C_29_H_50_O. The detailed structures of both molecules are presented in Figure 1.

This is the first study that explored the therapeutic significance of arbortristoside E. Thus, based on this study, arbortristoside E and beta-sitosterol could be suggested as potent anti-inflammatory drugs that can synergize anti-inflammatory effects by blocking different receptors.

## 2. Results and Discussion

### 2.1. Docking Results

In this study, we evaluated the potential anti-inflammatory binding affinity and interactions of the screened and selected compound Arb_E with different inflammatory target proteins that are involved in the mediation of inflammation, namely, COX-1, COX-2, PDE4, PDE7, IL-17A, IL-17D, TNF-α, IL-1β, prostaglandin E2, and prostaglandin F synthase. Additionally, these proteins are implicated in numerous pathological and physiological processes, including cell signaling, immunological responses, and pain. We used molecular docking to predict the binding energy and inhibition constant (Ki) of the compound with each receptor. We analyzed the hydrogen bonds and hydrophobic interactions formed between the compound and the target residues. The results are summarized in Table 1 and Table 2. Also, the docking poses of 3D representations of all receptors with Arb_E are provided in Supplementary Figure S1.

The control drug celecoxib, an NSAID, targets the cyclooxygenase-2 (COX-2) enzyme with a high affinity. The binding energy was observed to be −9.29 kcal/mol, which is indicative of a strong binding interaction. The inhibition constant (Ki) is 155.52 nM, which means that celecoxib can inhibit COX-2 activity at a concentration of 155.52 nM or less.

Table 1 also shows the presence of hydrogen bonds that form between celecoxib and COX-2. The first hydrogen bond is between the carboxylate group of the ARG513 residue in COX-2 and the hydroxyl group of the UNK0 residue in celecoxib. 

The hydrogen bond length is 2.04571 Å. The other hydrogen bond was observed to be between the carboxylate group of the ARG513 residue in COX-2 and the amide group of the UNK0 residue in celecoxib, and the observed hydrogen bond length was 3.00729 Å. Moreover, the carboxylate group of the ARG513 residue in COX-2 and the amine group of the UNK0 residue in celecoxib were also observed to be interacting with the involvement of hydrogen bonding with a bond length of 3.62419 Å (Table 1).

**Figure 2:**
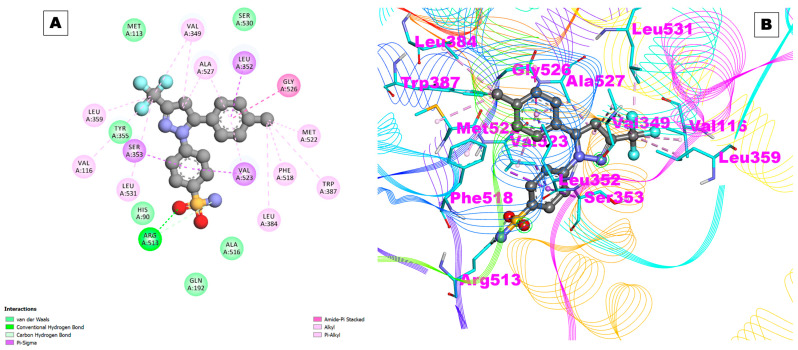
(**A**) Two-dimensional representation and (**B**) three-dimensional conformations of the interaction of the selected control drug celecoxib with COX-2.

The results of this study also indicated the involvement of hydrophobic interactions between celecoxib and COX-2. The amino acids Val116, Leu359, Tyr335, Ser353, Leu531, His90, Gln192, Ala516, Leu384, Val523, Phe518, Trp387, Met522, Gly526, Leu352, Ala527, Val349, Ser530, and Met113 were found to be interacting with hydrophobic residues (Figure 2A,B). Hydrophobic interactions are non-covalent interactions that occur between non-polar molecules. In this case, the hydrophobic residues on celecoxib interact with the hydrophobic residues on COX-2, which helps to stabilize the binding interaction.

Inhibitors of COX-1 and COX-2 are widely used as drug targets for anti-inflammatory and analgesic drugs [20].

**Figure 3:**
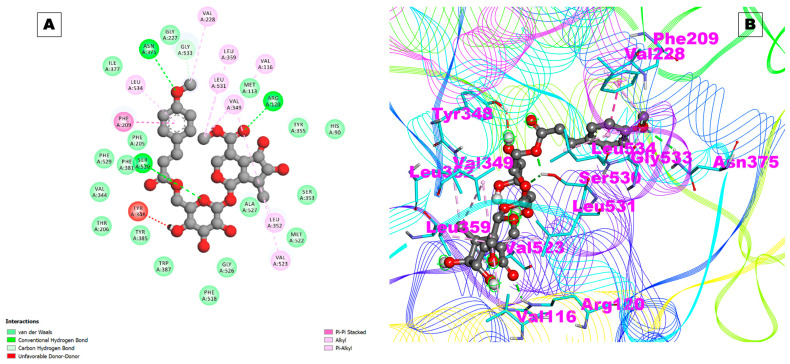
(**A**) Two-dimensional representation and (**B**) three-dimensional conformations of the interaction of the compound Arb_E with COX-2.

The Arb_E molecule showed a low binding affinity with COX-1 (−3.05 kcal/mol) and a Ki of 5.81 mM. The hydrogen bonds involved in the interactions were with the Tyr385 and Asn382 residues of COX-1, and two hydrophobic interactions were observed with the Phe210 and Pro400 amino acid residues. The docking results of the compound with COX-1 are given in Table 1. At the same time, a high binding affinity was observed with COX-2, with a binding energy of −10.26 kcal/mol and a significant inhibition constant (Ki:30.07 nM). Three hydrogen bonds with the Arg120, Asn375, and Ser530 residues of COX-2 and four hydrophobic contacts with the Gly533, Trp67, Pro110, and Pro164 residues were observed that could mediate the stable interactions to stabilize the complex (Figure 3A,B and Table 1).

The docking pose of Arb_E with COX-2 is shown in Figure 3A,B. The results of this study indicate that the studied compound inhibits COX-2 more potently than it inhibits COX-1, which is desirable for reducing the side effects associated with COX-1 inhibition, such as gastrointestinal irritation and ulcers. Our studied molecule, Arb_E, was also observed to have a high binding affinity with the proteins COX-2 and prostaglandin E2 (PGE2), which are the natural inflammatory mediators of COX-2. These interactions suggest that the studied molecule can synergize the anti-inflammatory action with PGE2 and bind to COX-2, resulting in reduced production.

PDE4 and PDE7 are enzymes that degrade cyclic adenosine monophosphate (cAMP), a secondary messenger that mediates many cellular responses. Inhibitors of PDE4 and PDE7 have been proposed as potential therapeutics for inflammatory diseases like asthma, chronic obstructive pulmonary disease (COPD), psoriasis, and rheumatoid arthritis [21].

Both molecules explored in this study were observed to have a high binding affinity with PDE4, with a binding energy of −9.00 kcal/mol and a Ki of 251.54 nM. The hydrogen bonds were observed to interact with the His416, Glu442, Asp484, and Gln555 residues of PDE4, and four hydrophobic residues were found to interact with the Ser420, Asp413, Pro400, and Leu401 residues. However, a moderate but significant binding affinity with PDE7 was observed, with a binding energy of −6.74 kcal/mol and a Ki of 11.43 uM. Four hydrogen bonds with the PDE7 residues Asp253, Glu282, Glu383, and Gln413 were found. Many studies have shown that bioactive components from natural plant extracts can inhibit PDE to treat a wide range of inflammatory conditions (Table 1). Many enticing plant-derived inhibitors have also been discovered, including coumarins (glycocoumarin and licoarylcoumarin), agapanthus saponins (saponin, lignin, kobusin, and (±)-schizandrin), terpenes (perianradulcin A, ursolic acid, and quinovic acid), anthraquinones (chrysophanol and emodin), and alkaloids (Sanjoinine-D) [22].

IL-17A and IL-17D are members of the IL-17 family of cytokines, which are involved in inflammation, immune responses, and cell signaling. IL-17A is the main cytokine of Th17 cells, which may contribute to a host’s ability to defend itself against infections and autoimmune illnesses. IL-17D is a less studied cytokine that is expressed by various tissues and cells and has been implicated in tumor suppression and viral infections [23].

The study of the IL interactions with phytochemicals showed significant interactions with both IL-17A and IL-17D (binding energies of −5.81 and −6.70 kcal/mol and Ki values of 54.74 and 12.36 uM, respectively). The molecules were observed to have two hydrogen bonds mediated through amino acids, namely, the Val65 and Tyr62 residues of IL-17A, and four hydrophobic contacts with the Leu53, Pro63, Trp67, and Gln94 residues of IL-17A. In comparison, IL-17D was observed to interact with the molecules through four hydrogen bonds via the Val141, Glu282, Glu383, and Gln413 residues of IL-17D and four hydrophobic contacts with the Ala78, Pro110, Pro164, and Cys402 residues (Table 1). The anti-inflammatory potentials of many phytochemicals that mediate their action through IL-17A and IL-17D have been evaluated, which supports the significance of this study. These findings support the idea that the natural chemical under investigation can bind to both IL-17A and IL-17D receptors and regulate the signaling pathways involved in an inflammatory response. More research is needed to determine the biological effects of our chemical on these cytokines. Both tumor necrosis factor-alpha and interleukin-1beta are pro-inflammatory cytokines that mediate various inflammatory and immune responses [19]. They are involved in diseases including rheumatoid arthritis, ulcerative colitis, Alzheimer’s disease, and Crohn’s disease. Inhibitors of TNF-α and IL-1β have been developed as anti-inflammatory drugs for these diseases [24]. 

Our compounds were observed to have significant interactions with TNF-α, with a binding energy of −4.70 kcal/mol and a Ki of 360.94 uM. The Leu142 and Gln67 residues of TNF-α were found to interact significantly via hydrogen bonds (binding energy of −4.59 kcal/mol and Ki of 431.16 uM), while no hydrogen bonds were formed between the compound and the receptor. The compound formed one hydrogen bond with the Lys103 residue of IL-1β and one hydrophobic contact with the Phe150 residue. These data show that while our drug is highly effective at blocking TNF-αand IL-1, it has only a modest effect on their anti-inflammatory properties. TNF-α and IL-1 interacted with the molecule less strongly than they did with their native ligands, TNF receptor 1 (TNFR1) and IL-1 receptor 1, respectively. This suggests that our chemical probably does not disrupt the normal signaling of these cytokines.

Prostaglandin F synthase (PGFS) is a member of the aldo-keto reductase (AKR) superfamily of enzymes. It catalyzes the conversion of prostaglandin D2 (PGD2) to prostaglandin F2α (PGF2α), which shows a binding energy of −10.19 kcal/mol and an inhibition constant of 33.66 nM. Also, 10 hydrogen bonds and the amino acid residues Thr251, Gln279,Asn280,ALA253,Arg276,Leu219,Leu236,Ala269,Leu268,Ala218,Ser221,Gly22,Tyr216,Thr23,Ser51,Lys84, and His117were involved in hydrophobic interactions (Table 1).

In this study, we also evaluated the binding affinity and interactions of beta-sitosterol, a plant sterol with cholesterol-lowering and anti-inflammatory properties, with the receptors COX-1, COX-2, PDE4, PDE7, IL-17A, IL-17D, TNF-α, IL-1β, prostaglandin E2, and prostaglandin F synthase. These receptors are involved in various physiological and pathological processes, such as inflammation, pain, immune responses, and cell signaling. The results are summarized in Table 2. Also, the docking poses of 3D representations of all receptors with Beta_sito are provided in Supplementary Figure S1.

The COX-1 and COX-2 enzyme proteins mediate the synthesis of prostaglandins from arachidonic acid. Prostaglandins are involved in inflammation, pain, fever, and other physiological processes. Inhibitors of cyclooxygenase-1 and -2 are commonly used as medicines for treating inflammation and pain. Beta_sito was observed to interact with a moderate binding affinity to COX-1 (−4.91 kcal/mol and a Ki of 253.57 uM). Beta_sito did not form any hydrogen bonds with COX-1 but interacted with many hydrophobic contacts with various residues of COX-1, which indicates complex stability.

**Figure 4:**
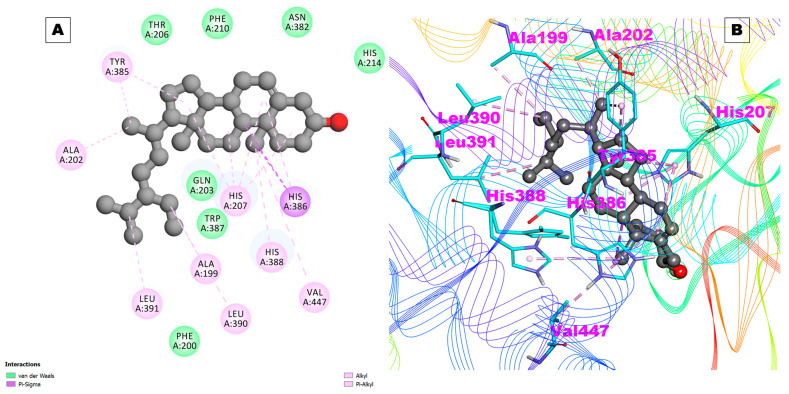
(**A**) Two-dimensional representation and (**B**) three-dimensional conformations of the interaction of the compound Beta_sito with COX-2.

Beta_sito was observed to interact with a high binding affinity with COX-2 (−8.86 kcal/mol and a Ki of 320.37 nM) (Table 2). Beta_sito also did not form any hydrogen bonds with COX-2, but it developed 18 hydrophobic contacts with different residues of COX-2. The docking pose of Beta_sito with COX-2 is shown in Figure 4A,B. These results indicate that Beta_sito is a selective inhibitor of COX-2 over COX-1, which is desirable for reducing the side effects associated with COX-1 inhibition, such as gastrointestinal bleeding and ulcers [25,26]. Beta_sito also showed a higher binding affinity with COX-2 than prostaglandin E2 (PGE2), which is the natural substrate of COX-2 [27]. This suggests that Beta_sito can compete with PGE2 to bind with COX-2 and reduce its production. PDE4 and PDE7 are enzymes that degrade cyclic adenosine monophosphate (cAMP), which is a second messenger that mediates various cellular responses. Inhibitors of PDE4 and PDE7 have been proposed as potential therapeutics for inflammatory diseases, such as rheumatoid arthritis, chronic obstructive pulmonary disease (COPD), asthma, and psoriasis [28]. Beta_sito showed a high binding affinity with PDE4, with a binding energy of −8.66 kcal/mol and a Ki of 448.17 nM (Table 2). Beta_sito did not form any hydrogen bonds with PDE4, but it formed 17 hydrophobic contacts with several residues of PDE4. Beta_sito also showed a high binding affinity with PDE7, with a binding energy of −7.48 kcal/mol and a Ki of 3.68 uM. Beta_sito interacted with two hydrogen bonds with the His256 and Asp253 residues of PDE7[29,30] (Table 2). IL-17A and IL-17D are members of the IL-17 family of cytokines, which are involved in inflammation, immune responses, and cell signaling. IL-17A is the signature cytokine of Th17 cells, which play a role in host defense against pathogens and autoimmune diseases [31].

IL-17D is a less studied cytokine that is expressed by various tissues and cells, and it has been implicated in tumor suppression and viral infections. Beta_sito showed a high binding affinity with IL-17A, with a binding energy of −7.43 kcal/mol and a Ki of 3.60 uM (Table 2). Beta_sito formed a hydrogen bond with the Trp67 amino acid residue of IL-17A and formed 11 hydrophobic contacts with different residues of IL-17A. Beta_sito also showed a high binding affinity with IL-17D, with a binding energy of −8.22 kcal/mol and a Ki of 947.55 nM (Table 2). Beta_sito formed a hydrogen bond with the Val165 residue of IL-17D and formed nine hydrophobic contacts with various residues of IL-17D (Table 2). These results suggest that Beta_sito can bind to both IL-17A and IL-17D receptors and modulate their signaling pathways. However, the biological effects of Beta_sito on these cytokines are unclear and need further investigation. TNF-α and IL-1β are pro-inflammatory cytokines that mediate various inflammatory and immune responses. They are involved in diseases, such as rheumatoid arthritis, Crohn’s disease, ulcerative colitis, and Alzheimer’s disease. Inhibitors of TNF-α and IL-1β have been developed as anti-inflammatory drugs for these diseases. Beta-sito showed a high binding affinity with TNF-α, with a binding energy of −7.10 kcal/mol and a Ki of 6.20 uM. Beta_sito formed one hydrogen bond with the Gln67 residue of TNF-α and formed 10 hydrophobic contacts with several residues of TNF-α. Beta_sito also showed a moderate binding affinity with IL-1β, with a binding energy of −6.19 kcal/mol and a Ki of 29.24 uM (Table 2). Beta-sito formed one hydrogen bond with the Met148 residue of IL-1β and formed nine hydrophobic contacts with different residues of IL-1β. These results indicate that Beta_sito is a potent inhibitor of TNF-α and a moderate inhibitor of IL-1β, and it may have beneficial effects that modulate their inflammatory actions. Beta_sito also showed a higher binding affinity with TNF-α and IL-1β than their natural ligands, TNF receptor 1 (TNFR1) and IL-1 receptor 1 (IL-1R1), respectively. This implies that beta-sitosterol may interfere with the normal signaling of these cytokines [32,33,34]. Prostaglandin F synthase (PGFS) showed a binding energy of −7.39 kcal/mol with an inhibition constant of 3.83 uM. No hydrogen bonds formed during the interaction. However, hydrophobic interactions were created by amino acid residues, namely, Arg223, Leu236, Gly220, Leu219, Ala218, Ala269, Tyr55, Ser217, Tyr55, Tyr24, Tyr216, Gly22, Asp50, Leu268, Thr23, Lys270, Gln222, and Ser221 (Table 2).

### 2.2. ADMET Results

Table S3 presents the results of the ADME predictions for three compounds using the Swiss ADME platform. ADME stands for absorption, distribution, metabolism, and excretion. These are the four main processes that determine how a drug is absorbed into the body, distributed to different tissues, metabolized by the liver, and excreted by the kidneys. Celecoxib, which has high GI absorption, is predicted to be able to cross the blood–brain barrier (BBB). It is also predicted to be a substrate for the P-glycoprotein (Pgp) transporter, which is an efflux transporter that can pump drugs out of cells. The compound is also predicted to be an inhibitor of CYP1A2, CYP2C19, and CYP2C9, which are three of the major cytochrome P450 enzymes that catalyze metabolism. Finally, the compound has a negative log Kp of −6.21, which indicates that it is poor at permeating skin. Arb_E, which has low GI absorption, is not predicted to be able to cross the BBB. It is also not predicted to be a substrate for Pgp or to inhibit any of the CYP enzymes. Finally, the compound has a negative log Kp of −9.95, which indicates that it is very poor at permeating skin. Beta-sito, which also has low GI absorption, is not predicted to be able to cross the BBB, be a substrate for Pgp, or inhibit any of the CYP enzymes. Finally, the compound has a negative log Kp of −2.2, which indicates that it is relatively good at permeating skin. These results can be used to help design drug molecules with desired properties. For example, if a drug that can cross the BBB is desired, then it is essential to avoid compounds that are predicted to be Pgp substrates or inhibitors of CYP enzymes. Similarly, if a drug that has good skin permeation is desired, then it is essential to avoid compounds with high negative log Kp values.

The drug-likeness predictions for the three compounds are given in Table S4. Celecoxib is a known drug that is used to treat pain and inflammation. It has a molecular weight of 381.37 g/mol, four rotatable bonds, seven hydrogen bond acceptors, one hydrogen bond donor, and a total polar surface area (TPSA) of 86.36 Å^2^. It has a consensus log P of 3.4, which is within the desired range for drug-like molecules. It does not violate any of the Lipinski, Ghose, Veber, Egan, or Muegge rules, which suggests that it is likely to be orally bioavailable and have good metabolic stability. It has a bioavailability score of 0.55, which is acceptable. Its synthetic accessibility is 2.74, suggesting it is relatively easy to synthesize.

To the best of our knowledge, Arb_E has not yet been tested in humans. It has a molecular weight of 566.55 g/mol, 10 rotatable bonds, 5 hydrogen bond acceptors, and 13 hydrogen bond donors. It has a total polar surface area of 190.67 Å^2^ and a consensus log P of −0.14. It violates the Lipinski rule for the number of hydrogen bond acceptors, but it does not violate any of the other rules. This suggests that it may have poor oral bioavailability and metabolic stability, supporting the observed low bioavailability score of 0.11. Its synthetic accessibility is 6.51, suggesting it is relatively difficult to synthesize. Beta-sitosterol is a naturally occurring compound that has been reported to have significant antioxidant and anti-inflammatory properties. It has a molecular weight of 414.71 g/mol, six rotatable bonds, one hydrogen bond acceptor, and a hydrogen bond donor. It has a total polar surface area of 20.23 Å^2^ and a consensus log P of 7.19. It violates the Lipinski rule for molecular weight, but it does not violate any of the other rules. This suggests that it may have poor oral bioavailability and metabolic stability. It has a bioavailability score of 0.55, which is low. Its synthetic accessibility is 6.3, suggesting it is relatively difficult to synthesize.

Overall, the drug-likeness predictions for the three compounds in Table S2 suggest that celecoxib is the most likely to be a successful drug. It has molecular weight, number of rotatable bonds, TPSA, and consensus log P values within the desired ranges. It does not violate any Lipinski, Ghose, Veber, Egan, or Muegge rules. It has a high bioavailability score and relatively easy synthetic accessibility. Arb_E and beta-sitosterol have some drug-like properties and could be tested to develop novel and potential anti-inflammatory molecules. The toxicity predictions for the three compounds are given in Table S5. Celecoxib is a known drug that is used to treat pain and inflammation. It is predicted to be mutagenic, but it is below the standard cut-off for toxicity. It has a maximum tolerated dose (MTD) of 0.021 log(mg/kg/day), which is below the standard cut-off for toxicity. It is not predicted to be an inhibitor of hERG I or hERG II, which are proteins involved in the heart’s electrical activity. It has an oral rat acute toxicity (LD50) value of 2.027, which is above the standard cut-off for toxicity. It has an oral rat chronic toxicity (LOAEL) value of 0.963, which is below the standard cut-off for toxicity. It is not predicted to be hepatotoxic or to cause skin sensitization. It is not predicted to be toxic to the nematode *T. pyriformis* or minnows. Arb_E is a potential molecule that has not yet been tested in humans. It is not predicted to be mutagenic. It has an MTD of −0.151 log(mg/kg/day), which is below the standard cut-off for toxicity. It is not predicted to inhibit hERG I or hERG II. It has an oral rat acute toxicity (LD50) value of 3.197, which is above the standard cut-off for toxicity. It has an oral rat chronic toxicity (LOAEL) value of 3.25, which is above the standard cut-off for toxicity. It is not predicted to be hepatotoxic or to cause skin sensitization. It is not predicted to be toxic to the nematode *T. pyriformis* or minnows. Beta-sitosterol is a naturally occurring compound that has been shown to have anti-inflammatory and antioxidant properties. It is not predicted to be mutagenic. It has an MTD of −0.621 log(mg/kg/day), which is below the standard cut-off for toxicity. It is not predicted to inhibit hERG I or hERG II. It has an oral rat acute toxicity (LD50) value of 2.552, which is above the standard cut-off for toxicity. It has an oral rat chronic toxicity (LOAEL) value of 0.855, which is below the standard cut-off for toxicity. It is not predicted to be hepatotoxic or to cause skin sensitization. It is not predicted to be toxic to the nematode *T. pyriformis* or minnows.

Overall, it was observed that celecoxib is the safest of the three compounds. It is below the standard cut-off for toxicity in all categories, but the studied molecules, Arb_E and beta-sitosterol, have some toxicity concerns. However, they are also below the standard cut-off for toxicity in most categories. It is important to note that these predictions are just that, predictions. The actual behavior of a drug in the body may differ from what is predicted. Therefore, conducting experimental studies to confirm the predictions is always essential.

### 2.3. Molecular Dynamics and Simulation Analysis

After successfully running the simulation, GROMACS version 2018 trajectory files were analyzed using the XMGRACE tool version 5.1. The 2D plots were analyzed for root-mean-square fluctuation (RMSF), root-mean-square deviation (RMSD), radius of gyration (RoG), and hydrogen bond formation during a 100 ns simulation. 

**Figure 5:**
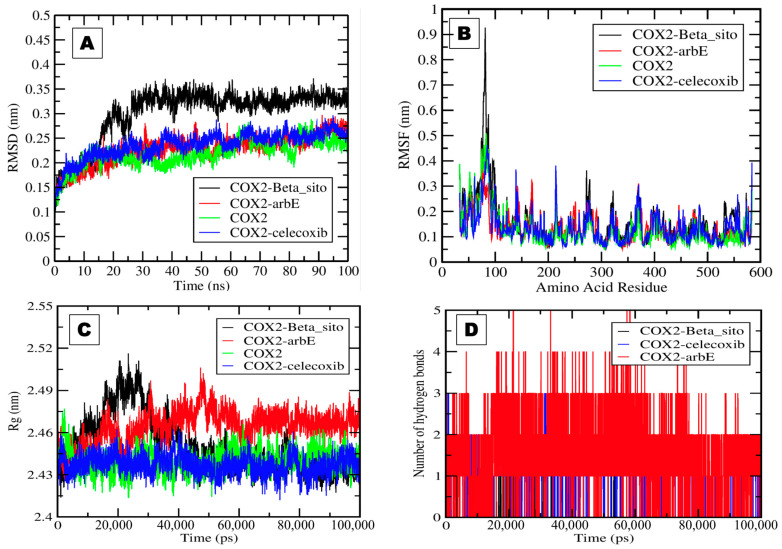
The plots generated by the trajectory file. (**A**) RMSD plot of COX-2–Beta_sito complex (black), COX-2–Arb_E complex (red), COX-2 in water (green), and COX-2–celecoxib complex (blue). (**B**) RMSF plot showing the fluctuation per amino acid residue. (**C**) The radius of gyration (Rg) plot represents the COX-2 tightening and compactness in the presence of Beta_sito, Arb_E, and celecoxib. (**D**) The plot shows the number of hydrogen bond interactions during a 100 ns simulation of selected complexes.

It was observed that the Beta_sito complex showed a higher value, i.e., 0.3 nm, than the other complexes with stable patterns. The deviation in the COX-2 simulation in water, the COX-2 simulation in the presence of Arb_E, and the control drug celecoxib ranged from 0.15 to 0.25 nm (Figure 5A). COX-2 and COX-2–Arb_E complexes showed average values of 0.2 nm. The complexes’ RMSF fluctuation plot values ranged from 0.1 to 0.9 nm. The average observed RMSF values for all selected complexes were very close to 0.1 nm, with some significant fluctuation in the 70–100 amino acid residue region (Figure 5B). The radius of gyration values represents the compactness and stability of the proteins’ tertiary structures. It was necessary to calculate these values to assess protein integrity in the presence of the selected compounds. The plot shows the radius of gyration values between 2.43 and 2.52 nm for all complexes. The COX-2 simulation in water and the presence of Beta_sito and celecoxib in the system showed approximately similar values near 2.46 nm. In comparison, Arb_E showed values slightly higher than 2.46 nm and showed a stable pattern except for some fluctuations between 20 and 40 ns (Figure 5C).

Hydrogen bonds play an essential role during ligand–protein interactions and are another assessable factor for the interaction and thermodynamic analysis of complexes [35]. We generated a hydrogen bond plot showing that the COX-2–Arb_E complex formed five hydrogen bonds, while the COX-2–Beta_sito complex formed only one bond. In contrast, the COX-2–celecoxib complex formed three hydrogen bonds during the simulation (Figure 5D).

### 2.4. MM-PBSA Results

In this study, we investigated the impact of Arb_E, Beta_sito, and celecoxib ligands on the COX-2–arbE, COX-2–Beta_sito, and COX-2–celecoxib complexes, respectively, through a comparative binding energy approximation, as presented in Table 3. Our findings reveal a distinct binding energy (ΔG_bind_) pattern, notably with Arb_E bound to COX-2 exhibiting lower binding energy of −277.602 kJ/mol compared to Beta_sito (−214.385 kJ/mol) and celecoxib (−193.635 kJ/mol) ligands bound with COX-2–beta_sito and COX-2–celecoxib complexes. Specifically, significant net fluctuations in the binding energy of −83.967 kJ/mol, −63.217 kJ/mol, and −20.75 kJ/mol were observed for Arb_E vs. celecoxib, Arb_E vs. Beta_sito, and Beta_sito vs. celecoxib, respectively, indicating a higher energy value for Arb_E and Beta_sito when bound to their respective receptor COX-2.

We evaluated the binding affinity and interactions of Beta_sito, a plant sterol with anti-inflammatory properties, with the receptors COX-1, COX-2, PDE4, PDE7, IL-17A, IL-17D, TNF-α, IL-1β, prostaglandin E2, and prostaglandin F synthase using molecular docking. We found that Beta_sito is a selective inhibitor of COX-2 over COX-1, a dual inhibitor of PDE4 and PDE7, a potent inhibitor of TNF-α, and a moderate inhibitor of IL-1β. Beta_sito also showed high binding affinities with IL-17A and IL-17D, but the biological effects of Beta_sito on these cytokines are unclear. Beta_sito may have potential applications for treating inflammatory diseases, but further studies are needed to confirm its biological effects.

## 3. Materials and Methods

### 3.1. Ligand Preparation

Nonsteroidal anti-inflammatory drugs (NSAIDs) selectively block the COX-2 enzyme, which regulates prostaglandin synthesis. This is because inflammation causes an increase in the production of the prostaglandin enzyme COX-2. Because of their efficacy in alleviating pain and preventing inflammation-related disorders, selective COX-2 inhibitors have been a key focus of anti-inflammatory medication development. Improved toxicity in the gut and fewer side effects are just two ways that such blockers excel compared to standard NSAIDs. Thus, in this study, the selective COX-2 inhibitor control drug celecoxib was selected to evaluate the potency of the anti-inflammatory effects using phytochemicals. We selected the two best compounds, arbortristoside E (Arb_E) and beta-sitosterol (Beta-sito), based on binding affinity during a preliminary virtual screening of 26 natural compounds *from Nyctanthes arbor-tristis* (Supplementary Tables S1 and S2).

We obtained the 2D structures and SMILES IDs of the key natural compounds of *Nyctanthes arbor-tristis* and the control drug celecoxib from the PubChem database (https://pubchem.ncbi.nlm.nih.gov/) (accessed on 11 October 2023) [36]. We then used the Novoprolab server (https://www.novoprolabs.com/tools/smiles2pdb) (accessed on 12 October 2023) to convert the SMILES IDs into 3D PDB files [37] for subsequent molecular docking and simulation studies. Next, we submitted the ligand files to Discovery Studio Visualizer version 21.1.0.20298 for the energy minimization [38]. We applied the CHARMm force field to model the macromolecular systems using the empirical energy functions [39,40].

### 3.2. Receptor Preparation

The COX-2 receptor is a protein that catalyzes the synthesis of prostaglandins, which are involved in inflammation and pain. The crystal structure of the human COX-2 receptor (PDB ID: 5F1A) was obtained from the PDB database (https://www.rcsb.org/structure/5F1A) (accessed on 19 October 2023) [41]. 

In the next step, HOH atoms and HETATM were deleted from the native PDB files, and the CHARMm force field was used to minimize energy using Discovery Studio Visualizer version 21.1.0.20298 [39].

This step involved finding the receptor region where the ligand could bind and interact with its amino acid residues. We analyzed the binding site of 5F1A in Discovery Studio Visualizer version 21.1.0.20298 [42]. The key amino acid was identified and considered for the active site docking of selected natural compounds.

### 3.3. AutoDock 4.2 Tool Receptor–Ligand Docking

In AutoDock 4.2, water molecules, cofactors, and other unwanted molecules from the receptor structure were removed, and the addition of hydrogen atoms, assigning charges and atom types, and optimizing the geometry were performed. For the ligand, we needed to generate different conformations and tautomers that could fit into the receptor binding site.

Furthermore, we set up a grid box that covered the region of interest where the docking was performed. The grid box defined the size and resolution of the grid points used to calculate the interaction energies between the receptor and the ligand [43]. The grid points x, y, and z were set as 60 × 60 × 60, and the spacing was 0.375 A. The center-grid x,y, and z co-ordinates were −36.659, −51.728, and 2.072 (COX-1); 41.585, 25.501, and 237.603 (COX-2); 96.897, 66.877, and 19.023 (PDE4); 0.0192, 49.098, and 20.118 (PDE7); 79.997, −40.055, and −38.557 (IL-17A); 37.218, −35.236, and 4.499 (IL-17D); 24.212, 63.416, and 44.088 (TNF-α); 11.193, 20.924, and −9.834 (IL-1β); and −45.708, −42.479, and 0.232 (prostaglandin E2).

After that, the docking algorithm, the scoring function, and the output options were set to their default parameters. The docking Lamarckian genetic algorithm (LGA) and an empirical binding free energy function determined how the ligand was placed and rotated in the receptor binding site. The scoring function evaluated how well the ligand fits into the receptor and estimated its binding affinity (ΔG) according to the following formula:

Δ*G*_binding_ = Δ*G*_gauss_ + Δ*G*_repulsion_ + Δ*G*_hbond_ + Δ*G*_hydrophobic_ + Δ*G*_tors_,

where Δ*G*_gauss_ is an attractive term for the dispersion of two Gaussian functions; Δ*G*_repulsion_ is the square of the distance if it is closer than a threshold value; Δ*G*_hbond_ is a ramp function that is also used for interactions with metal ions; Δ*G*_hydrophobic_ is a ramp function; and Δ*G*_tors_ is proportional to the number of rotatable bonds [44].

Finally, the AutoDock 4.2 program executed the provided parameters after a successful run and prepared receptor and ligand files, the defined docking grid, and the parameters. Depending on the size and complexity of the receptor and the ligand [45], the population size (ga_pop_size), energy evaluations (ga_num_generation), mutation rate, crossover rate, and step size were set to 150, 2500,000, 27,000, 0.02, 0.8, and 0.2. The LGA runs were limited to 10 runs.

The last examination of the docking poses, ranking them according to their scores, visualizing them using molecular graphics software, and comparing them with control data, was completed using Discover Studio Visualizer version 20.1.0.19295 [42,46].

### 3.4. Drug-Likeness and ADMET

We used the SwissADME online tool (http://www.swissadme.ch) (accessed on 25 October 2023) from the Swiss Institute of Bioinformatics (SIB), Lausanne, Switzerland [47,48,49], to computationally predict the ADME, drug-likeness, and pharmacokinetic properties of the selected natural compounds. We also analyzed additional toxicity using the pkCSM online server (http://biosig.unimelb.edu.au/pkcsm/) (accessed on 25 October 2023) [50].

### 3.5. MDS

We performed a 100 ns MDS of the selected complexes of COX-2–Arb_E, COX-2–Beta-sito, and COX-2–celecoxib using the GROMACS version 2018 computational software [51]. 

**Figure 6:**
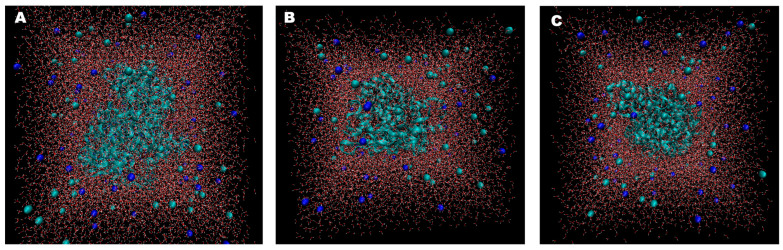
Three-dimensional visualizations of solvated and neutralized states of (**A**) COX-2–Arb_E, (**B**) COX-2–Beta_sito, and (**C**) COX-2–celecoxib. The complexes are shown as cyan ribbon patterns in the centers, and NA^+^ (blue) and Cl^−^ (cyan) ions are round shapes in the surrounding water. The complexes were visualized using the VMD (Visual molecular dynamics) tool version 1.9.4a57 [58].

We also simulated COX-2 in water for comparison. Topology information for COX-2 was generated using the pdb2gmx package, and the CHARMM27 all-atom force field was utilized. We obtained the topology files of the ligands Arb_E, Beta-sito, and celecoxib from the SwissParam server [52]. We created a triclinic box unit cell filled with water for solvation and added Na^+^ and Cl^−^ ions to stabilize the system. In total, 59 Na^+^ ions and 61 Cl^−^ ions were used for neutralization, with a concentration of 0.15 molar. Also, the total numbers of molecules in the solvated box were 18,793, 18,788, and 18,503 for the COX-2–beta-sito, COX-2–Arb_E, and COX-2–celecoxib complexes, respectively (Figure 6A–C) [53].

We conducted energy minimization and then equilibrated the system using two-step ensembles, namely, NVT and NPT. The steepest descents are set to 5000 steps. These ensembles controlled and stabilized the temperature and pressure of the system throughout the simulation [54].

The equilibrium parameters for the simulation were a temperature of 300 K, a pressure of 1.0 bar, and an equilibration time of 100 ps. We used gmx rms to calculate the RMSD [55], gmx rmsf to calculate the RMSF, gmx gyrate to calculate the Rg [56], and gmx hbond to analyze the number of hydrogen bonds formed at the time of interaction. We used the XMGRACE version 5.1 program to generate 2D plots [57].

### 3.6. Molecular Mechanics-Poisson–Boltzmann Surface Area (MM-PBSA)

Kumari et al. [59,60] employed an approximation in their research article to calculate the binding free energies of selected complexes. This approximation is based on the Molecular Mechanics-Poisson–Boltzmann Surface Area (MM-PBSA) method [61] and utilizes a program designed for analyzing solvation properties in biomolecules, including proteins and complex systems. The approximation combines two components in calculating free energy, excluding the entropic contribution. The first component involves the potential energy in a vacuum (ΔG_MM_), which includes bonded terms (such as bond, angle, and torsion energies) and non-bonded terms like van der Waals (ΔG_VDW_) and electrostatic interactions (ΔG_Coulomb_). The second component (ΔG_Solvation_) considers solvation effects, incorporating the sum of two energy terms: polar (ΔG_Polar_) and non-polar (ΔG_Nonpolar_) solvation energies. The calculation employs an implicit solvation model [59]. In the MM-PBSA framework, the expression for the free energy method is as follows:

ΔG = ΔG_MM_ + ΔG_solvation_ (1)

where

ΔG_MM_ = ΔG_Electrostatic pot_ + ΔG_VDW_ (2)

The solvation energy is the amount of energy necessary to transfer a solute from the void to the solvent and is expressed as the sum of the polar and nonpolar energies (see Equation (3)).

ΔG_Solvation_ = ΔG_Polar_ + ΔG_nonpolar_ (3)

The polar term is intricately linked to the development of permanent dipoles. Conversely, the polar surface comprises permanent dipoles and is associated with the charge distribution of the solute. In our MM-PBSA calculation, the ionic strength was modified by adding 0.150 M of NaCl. The configuration parameters included setting the number of grid points per A2 to 10, and the maximum number of iterations for the linear Poisson–Boltzmann solver was established at 50,000.

## 4. Conclusions

The search for drugs and their development, particularly in anti-inflammatory studies, has significantly benefited from the in silico approach. The complicated nature of biological networks makes it challenging to describe and forecast the effects of medications on them. At the same time, there is a lack of experimental data to confirm computerized models. It is also possible that some compounds or targets need to be amenable to computational research. The computational technique has helped find and develop hit molecules, moving them further in the pipeline for drug discovery or into the market despite these constraints. By combining more experimental data and creating new approaches, researchers are constantly attempting to improve the precision and reliability of the in silico models that are used.

We used molecular docking to check how well a new compound binds to and interacts with the following receptors: COX-1, COX-2, PDE4, PDE7, IL-17A, IL-17D, TNF-α, IL-1β, prostaglandin E2, and prostaglandin F synthase. Our compound selectively blocks COX-2 over COX-1, blocks both PDE4 and PDE7, and binds to IL-17A and IL-17D to a moderate degree. However, our compound showed low binding affinities with TNF-α and IL-1β and may not affect their inflammatory signaling. Our compound may have potential applications for the treatment of inflammatory diseases. A future study is required to validate the results based on the current computational findings. Therefore, studied natural compounds could be potential anti-inflammatory molecules and need further in-vitro/in-vivo experimentation for developing novel anti-inflammatory drugs. The pharmaceutical industry could utilize the presented data to develop anti-inflammatory drugs.

## Figures and Tables

**Table 1:** The docking analysis data of Arb_E and selected receptors COX-1, COX-2, PDE4, PDE7, IL-17A, IL-17D, TNF-α, IL-1β, prostaglandin E2, and prostaglandin F synthase.

Receptors	PDB ID	Binding Energy (kcal/mol)	Inhibition Constant (Ki)	Hydrogen Bond Details	Hydrogen Bond Length (Angstrom)	Residues Involved in Hydrophobic Interaction
Control (Celecoxib)	Cox-2	−9.29	155.52 nM	A:ARG513:HH11—:UNK0:O	2.04571	Val116,Leu359,Tyr335,Ser353,Leu531,His90,Gln192,ALa516,Leu384,Val523,Phe518,Trp387,Met522,Gly526,Leu352,Ala527,Val349,Ser530,Met113
				A:ARG513:CD—:UNK0:O	3.00729	
				A:ARG513:CD—:UNK0:N	3.62419	
COX-1	6Y3C	−3.05	5.81 mM	A:TRP387:HN—:UNK1:O36	1.64895	Ala199,Ala202,Gln203,Thr206,His207,Phe210,Phe381,Asn382,Tyr385,His386,Trp387,His388,Leu390,Met391,Ile444
				UNK1:H49—A:ASN382:O	2.56551	
				UNK1:H50—A:ASN382:OD1	2.53999	
				UNK1:H71—A:TYR385:O	1.92435	
				UNK1:H70—A:TYR385:O	2.23812	
				A:HIS388:CA—:UNK1:O23	3.75406	
				UNK1:C40—A:PHE210:O	3.26066	
COX-2	5F1A	−10.26	30.07 nM	A:ARG120:HE—:UNK1:O38	2.11946	Val116,Arg120,Phe205,Phe209,Gly227,Val228,Tyr348,Val349,Leu352,Ser353,Tyr355, Leu359,Asn375,Ile377,Phe381,Tyr385,Trp387,Phe518,Met522,Val523,Gly526,Ala527,Phe529,Ser530,Leu531,Gly533,Leu534
				A:ASN375:HD22—:UNK1:O32	2.79305	
				A:SER530:HG—:UNK1:O19	2.24954	
				A:SER530:HG—:UNK1:O21	1.89473	
				UNK1:C33—A:GLY533:O	2.89113	
PDE4	2QYK	−9.00	251.54 nM	A:HIS416:HE2—:UNK1:O38	2.03017	Asp413,His416,Ser420,Asn421,Gln422,Leu441,Glu442,His445,Asp484,Met485,Ser486,Asn533,Thr545,Ile548,Phe552,Gln555,Ser567,Met569,Gln581,Phe584
				A:GLN555:HE22—:UNK1:O36	2.64094	
				UNK1:H49—A:ASP484:OD1	1.91855	
				UNK1:H50—A:GLU442:OE2	1.92009	
				A:SER420:CB—:UNK1:O13	3.0371	
				UNK1:C40—A:ASP413:OD1	3.165	
PDE7	1ZKL	−6.74	11.43 uM	A:HIS256:HE2—:UNK1:O34		Tyr211,His212, His216,Asp253,His256,Gly258,Asn260,Gln261,Leu281,Glu282,His285,Ile323,Asp362,Asn365,Trp376,Ser377,Val380,Glu383,Phe384,Gln387,Pro400,Leu401,Cys402,Gln413,Phe416
				A:HIS256:HE2—:UNK1:O35	3.03088	
				A:GLN413:HE22—:UNK1:O32	2.10147	
				UNK1:H69—A:ASP253:OD1	2.53525	
				UNK1:H71—A:GLU282:OE2	2.16071	
				UNK1:H49—A:GLU383:OE2	2.69909	
				UNK1:C40—A:PRO400:O	2.37041	
IL-17A	5HI4	−5.81	54.74 uM	UNK1:H50—A:VAL65:O	1.98348	Leu53,Tyr62,Pro63,Val65,Ile66,Trp67,Gln94,Ile96,Leu97,Val98,Leu99,Val117,Ser118,Val119
				UNK1:H69—A:TYR62:O	2.4814	
				A:PRO63:CD—:UNK1:O35	2.97263	
				A:VAL119:CA—:UNK1:O32	3.01728	
				UNK1:C33—A:TRP67:O	3.37552	
IL-17D	Modeled from MODBASE server	−6.70	12.36 uM	UNK1:H69—A:VAL141:O	2.64013	Ala78,Arg80,Tyr96,Tyr105,Pro106,Tyr108,Leu109,Pro110,Ala112,Thr140,Val141,Val142,Ile163,Pro164,Val165
				UNK1:C40—A:ALA78:O	2.95779	
				UNK1:C33—A:PRO110:O	3.29094	
				UNK1:C33—A:PRO164:O	3.0518	
TNF-α	1A8M	−4.70	360.94 uM	UNK1:C20—A:LEU142:O	3.23102	Pro20,Ala22,Gly24,Lys65,Gly66,Gln67,Asp140,Leu142,Phe144,Ala145
				UNK1:C33—A:GLN67:OE1	3.04695	
IL-1β	6Y8M	−4.59	431.16 uM	A:LYS103:HZ2—:UNK1:O7	2.66882	Lys103,Asn108,Lys109,Leu110,Phe146,Thr147,Met148,Gln149,Phe150
				UNK1:C33—A:PHE150:O	3.21082	
Prostaglandin E2	4YHL	−7.23	4.98 uM	UNK1:H49—A:THR168:OG1	2.63075	Ile23,Pro24,Met27,Val72,Val75,Thr76,Thr79,Tyr80,Leu99,Thr168,Trp169,Cys170,Arg316,Ser319,Val320
				UNK1:H50—A:THR168:O	1.8278	
				UNK1:C40—A:CYS170:O	3.05882	
Prostaglandin F synthase	1RY0	−10.19	33.66 nM	A:TYR24:HN—:UNK1:O7	2.28139	Thr251,Gln279,Asn280,ALA253,Arg276,Leu219,Leu236,Ala269,Leu268,Ala218,Ser221,Gly22,Tyr216,Thr23,Ser51,Lys84,His117
				A:SER217:HN—:UNK1:O36	2.31396	
				A:LYS270:HN—:UNK1:O35	2.95418	
				A:LYS270:HZ2—:UNK1:O23	2.7668	
				A:LYS270:HZ3—:UNK1:O23	2.84067	
				UNK1:H49—A:ASP50:OD2	2.45111	
				UNK1:H49—A:ASP50:O	2.87918	
				UNK1:H50—A:TYR55:OH	2.08453	
				UNK1:C20—A:LYS270:O	2.79899	
				UNK1:C33—A:THR251:OG1	3.62729	

**Table 2:** The docking analysis data of Beta_sito and selected receptors COX-1, COX-2, PDE4, PDE7, IL-17A, IL-17D, TNF-α, IL-1β, prostaglandin E2, and prostaglandin F synthase.

Receptors	PDB ID	Binding Energy (kcal/mol)	Inhibition Constant (Ki)	Hydrogen Bond Details	Hydrogen Bond Length (Angstrom)	Residues Involved in Hydrophobic Interaction
Control (Celecoxib)	Cox-2	−9.29	155.52 nM	A:ARG513:HH11—:UNK0:O	2.04571	Val116,Leu359,Tyr335,Ser353,Leu531,His90,Gln192,ALa516,Leu384,Val523,Phe518,Trp387,Met522,Gly526,Leu352,Ala527,Val349,Ser530,Met113
				A:ARG513:CD—:UNK0:O	3.00729	
				A:ARG513:CD—:UNK0:N	3.62419	
COX-1	6Y3C	−4.91	253.57 uM	NA	NA	Ala199,Phe200,Ala202,Gln203,Thr206,His207,Leu295,Tyr385,His386,Trp387,His388, Leu390,Met391,Tyr404,Leu408,Ile444
COX-2	5F1A	−8.86	320.37 nM	NA	NA	Ala199,Ala202,Gln203,Thr206,His207,Phe210,His214, Asn382,Tyr385,His386,Trp387,His388,Leu390,Leu391
PDE4	2QYK	−8.66	448.17 nM	NA	NA	Tyr371,His372,Asp413,His416,Asn421,Leu441,Glu442, His445,Thr483,Met485,Asp530,Leu531,Ile548,Phe552,Met569,Phe584,Ile588
PDE7	1ZKL	−7.48	3.68 uM	A:HIS256:HE2—:UNK1:O25	2.68409	Tyr211,His212,His216,His252,Asp253,His256,Leu281,Glu282,His285,Thr321,Ile323,Asp362,Val380,Phe384,Leu401,Gln413,Phe416,Leu420
				UNK1:H67—A:ASP253:OD1	2.14352	
IL-17A	5HI4	−7.43	3.60 uM	A:TRP67:HN—:UNK1:O25	2.23528	Tyr62,Pro63,Ile66,Trp67,Ile96,Leu97,Val98,Leu99,Leu112, Val117
				UNK1:H67—A:TRP67:O	1.86307	
IL-17D	Modeled from MODBASE server	−8.22	947.55 nM	UNK1:H67—A:VAL165:O	2.01343	Arg80,Arg81,Phe82,Trp94,Tyr96,Pro106,Tyr108,Pro110,Val165
TNF-α	1A8M	−7.10	6.20 uM	UNK1:H67—A:GLN67:OE1	2.0408	Pro20,Lys65,Gly66,Gln67,Asp140,Tyr141,Leu142,Asp143,Phe144,Ala145
IL-1β	6Y8M	−6.19	29.24 uM	UNK1:H67—A:MET148:O	2.23593	Leu6,Met44,Phe46,Lys103,Glu105,Asn108,Leu110,Thr147,Met148,Gln149,Phe150
Prostaglandin E2	4YHL	−8.58	514.41 nM	NA	NA	Ile23,Pro24,Met27,Thr69,Val72,Ser73,Thr76,Tyr80,Arg316,Ser319,Val320
Prostaglandin F synthase	1RY0	−7.39	3.83 uM	NA	NA	Arg223,Leu236,Gly220,Leu219,Ala218,Ala269,Tyr55,Ser217,Tyr55,Tyr24,Tyr216,Gly22,Asp50,Leu268,Thr23,Lys270,Gln222,Ser221

**Table 3:** Binding free energy components of selected complexes obtained from MM-PBSA analysis.

S.N0.	Ligands	Van der Wall Energy (kJ/mol)	Electrostatic Energy (kJ/mol)	Polar Salvation Energy (kJ/mol)	SASA Energy (kJ/mol)	Binding Energy (kJ/mol)
1.	Arb_E	−300.730 +/−13.113	−22.633 +/−9.119	47.260 +/−188.830	−28.499 +/−0.977	−277.602 +/−39.964
2.	Beta_sito	−232.379 +/−11.525	−0.160 +/−2.069	39.170 +/−34.210	−21.016 +/−1.008	−214.385 +/−36.906
3.	Celecoxib	−220.267 +/−0.184	−59.621 +/−0.124	103.2250 +/−0.94	−16.972 +/−0.011	−193.635 +/−0.573

## Data Availability

Data is contained within the article and Supplementary Material.

## Supplementary Materials

The following supporting information can be downloaded at: https://www.mdpi.com/article/10.3390/ph17010018/s1, Table S1: List of natural compounds of Nyctanthes arbor-tristis Linn and their chemical information extracted from PubChem Database. Table S2: Virtual screening data 26 natural compounds including control drug celecoxib interaction with COX-2. Data obtained from PyRx tool. Table S3: ADME prediction from SwissADME (GI = Gastro intestinal, BBB = Blood Brain Barrier, Pgp = P glycoprotein, CYP = Cytochrome, log Kp = skin permeation). Table S4. Drug-likeness prediction from SwissADME server (MW = Molecular Weight, TPSA = total polar surface area, Consensus Log P = average of all predicted Log Po/w. Table S5: toxicity prediction. Data obtained from pkCSM server. Figure S1: List of best docked poses of all selected receptors and their interaction with Arbortristoside-E. Figure S2: List of best-docked poses of all selected receptors and their interaction with Beta-sitosterol.
