# Supplementary material for: Computational Molecular Docking and Simulation-Based Assessment of Anti-Inflammatory Properties of Nyctanthes arbor-tristis Linn Phytochemicals
Source: Pharmaceuticals (Basel). 2023 Dec 22;17(1):18. doi: 10.3390/ph17010018 (PMC10820488; doi:10.3390/ph17010018)
Supplement: Supplementary file 1 [file pharmaceuticals-17-00018-s001.zip › pharmaceuticals-2729553-supplementary.pdf]

Table S1: List of natural compounds of *Nyctanthes arbor-tristis Linn* and their chemical information extracted from PubChem Database

S.No.	Compound Name	PubChem ID	Molecular Weight	Molecular formula	2D structure	SMILES ID
1.	methyl salicylate	4133	$C_8H_8O_3$	152.15 g/mol	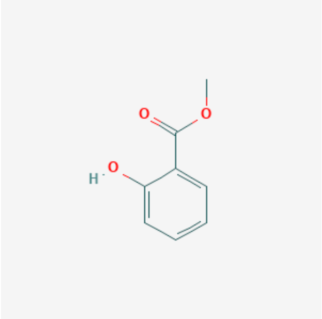	COC(=O)C1=CC=CC=C1O
2.	arborside-A	182902	$C_{31}H_{34}O_{13}$	614.6 g/mol	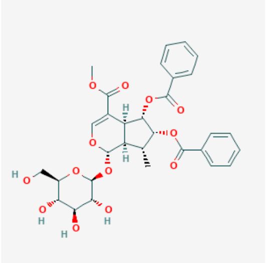	CC1C2C(C(C1OC(=O)C3=CC=CC=C3)OC(=O)C4=CC=CC=C4)C(=COC2OC5C(C(C(C(O5)CO)O)O)O)C(=O)OC
3.	Arborside-B	182903	$C_{24}H_{30}O_{11}$	494.5 g/mol	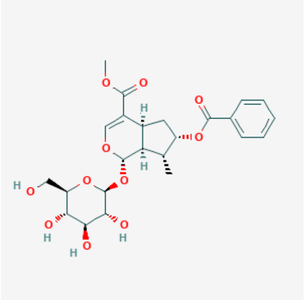	CC1C(CC2C1C(OC=C2C(=O)OC)OC3C(C(C(C(O3)CO)O)O)O)OC(=O)C4=CC=CC=C4

4.	Mannitol	6251	$C_6H_{14}O_6$	182.17 g/mol	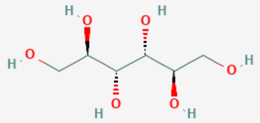	C(C(C(C(C(CO)O)O)O)O)O
5.	beta-Amyrin	73145	$C_{30}H_{50}O$	426.7 g/mol	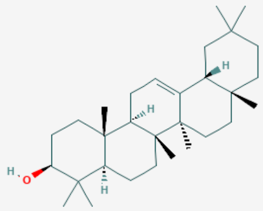	CC1(CCC2(CCC3(C(=CCC4C3(CCC5C4(CCC(C5(C)C)O)C)C)C2C1)C)C)C
6.	beta-Sitosterol	222284	$C_{29}H_{50}O$	414.7 g/mol	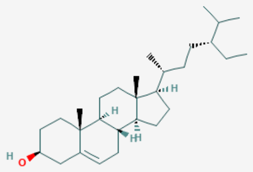	CCC(CCC(C)C1CCC2C1(CCC3C2CC=C4C3(CCC(C4)O)C)C)C(C)C
7.	hentriacontane	12410	$C_{31}H_{64}$	436.8 g/mol		CCCCCCCCCCCCCCCCCCCCCCCCCCCCCCC

8.	benzoic acid	243	$C_7H_6O_2$	122.12 g/mol	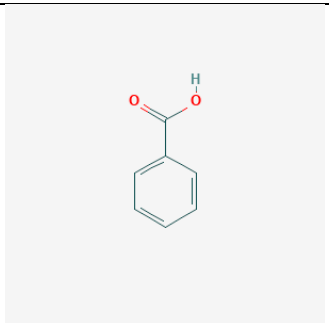	C1=CC=C(C=C1)C(=O)O
9.	astragalin	5282102	$C_{21}H_{20}O_{11}$	448.4 g/mol	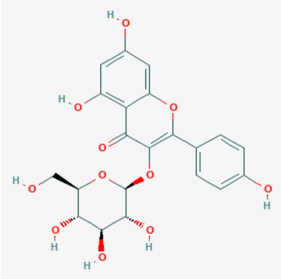	C1=CC(=CC=C1C2=C(C(=O)C3=C(C=C(C=C3O2)O)O)OC4C(C(C(C(O4)CO)O)O)O)O
10.	nicotiflorin	5318767	$C_{27}H_{30}O_{15}$	594.5 g/mol	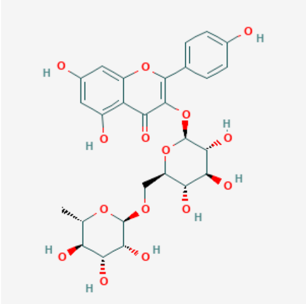	CC1C(C(C(C(O1)OCC2C(C(C(C(O2)OC3=C(OC4=CC(=CC(=C4C3=O)O)O)C5=CC=C(C=C5)O)O)O)O)O)O)O
11.	Nyctanthic acid	12313631	$C_{30}H_{48}O_2$	440.7 g/mol	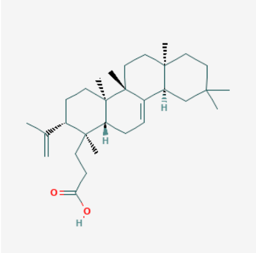	CC(=C)C1CCC2(C(C1(C)CCC(=O)O)CC=C3C2(CCC4(C3CC(CC4)(C)C)C)C)C

12.	lupeol	259846	$C_{30}H_{50}O$	426.7 g/mol	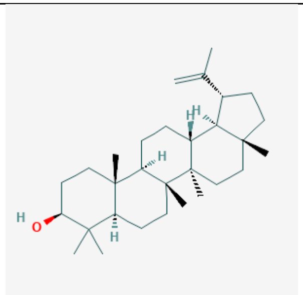	CC(=C)C1CCC2(C1C3CCC4C5(CCC(C(C5CCC4(C3(CC2)C)C)(C)C)O)C)C
13.	Alpha-xylopyranoside	3080780	$C_{11}H_{20}O_{10}$	312.27 g/mol	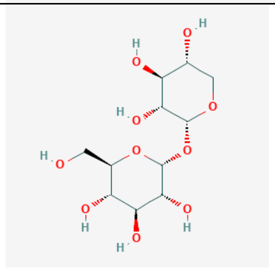	C1C(C(C(C(O1)OC2C(C(C(C(O2)CO)O)O)O)O)O)O
14.	Arbortristoside-B	5459045	$C_{26}H_{32}O_{15}$	584.5 g/mol	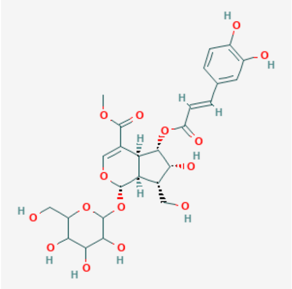	COC(=O)C1=COC(C2C1C(C(C2CO)O)OC(=O)C=CC3=CC(=C(C=C3)O)O)OC4C(C(C(C(O4)CO)O)O)O
15.	Arbortristoside C	23955893	$C_{26}H_{32}O_{13}$	552.5 g/mol	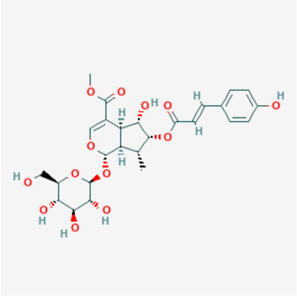	CC1C2C(C(C1OC(=O)C=CC3=CC=C(C=C3)O)O)C(=COC2OC4C(C(C(C(O4)CO)O)O)O)C(=O)OC

16.	arbortristoside-D	14632886	$C_{26}H_{32}O_{15}$	584.5 g/mol	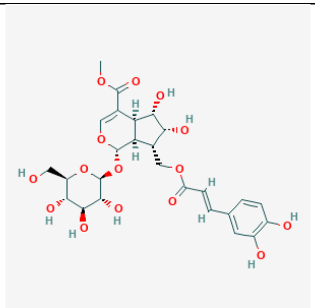	COC(=O)C1=COC(C2C1C(C(C2COC(=O)C=CC3=CC(=C(C=C3)O)O)O)O)OC4C(C(C(C(O4)CO)O)O)O
17.	Arbortristoside-E	14632884	$C_{27}H_{34}O_{13}$	566.5 g/mol	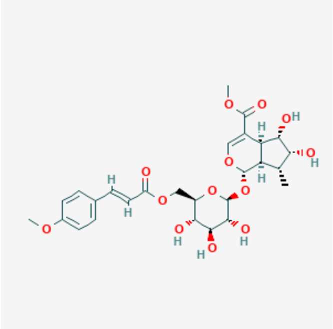	CC1C2C(C(C1O)O)C(=COC2OC3C(C(C(C(O3)COC(=O)C=CC4=CC=C(C=C4)OC)O)O)O)C(=O)OC
18.	6beta-Hydroxyloganin	158641	$C_{17}H_{26}O_{11}$	406.4 g/mol	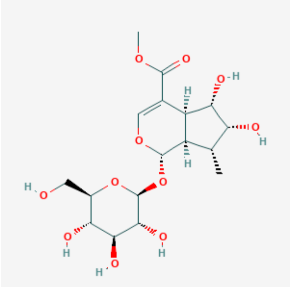	CC1C2C(C(C1O)O)C(=COC2OC3C(C(C(C(O3)CO)O)O)O)C(=O)OC

19.	Arborside-C	182904	$C_{24}H_{30}O_{12}$	510.5 g/mol	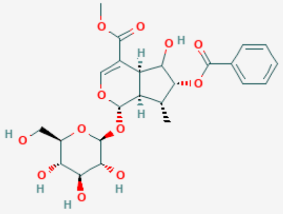	CC1C2C(C(C1OC(=O)C3=CC=CC=C3)O)C(=COC2OC4C(C(C(C(O4)CO)O)O)O)C(=O)OC
20.	p-cymene	7463	$C_{10}H_{14}$	134.22 g/mol	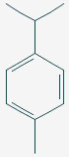	CC1=CC=C(C=C1)C(C)C
21.	1-deconol	8174	$C_{10}H_{22}O$	158.28 g/mol	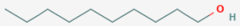	CCCCCCCCCCO
22.	alpha-Pinene	6654	$C_{10}H_{16}$	136.23 g/mol	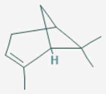	CC1=CCC2CC1C2(C)C

23.	Apigenin	5280443	$C_{15}H_{10}O_5$	270.24 g/mol	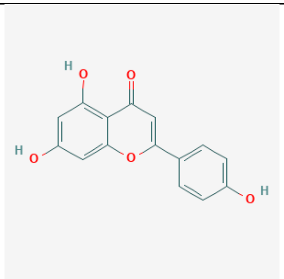	C1=CC(=CC=C1C2=CC(=O)C3=C(C=C(C=C3O2)O)O)O
24.	Kaemferol	5280863	$C_{15}H_{10}O_6$	286.24 g/mol	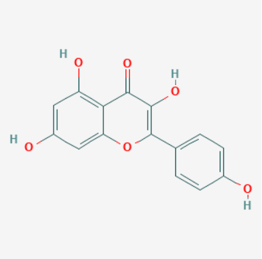	C1=CC(=CC=C1C2=C(C(=O)C3=C(C=C(C=C3O2)O)O)O)O
25.	Quercetin	5280343	$C_{15}H_{10}O_7$	302.23 g/mol	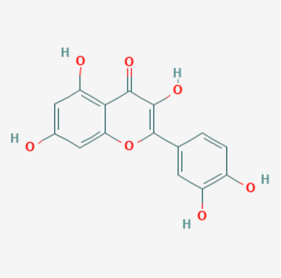	C1=CC(=C(C=C1C2=C(C(=O)C3=C(C=C(C=C3O2)O)O)O)O)O
26.	Nyctanthoside	95224501	$C_{17}H_{26}O_{12}$	422.4 g/mol	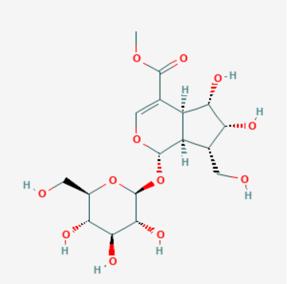	COC(=O)C1=COC(C2C1C(C(C2CO)O)O)OC3C(C(C(C(O3)CO)O)O)O

Table S2: Virtual screening data 26 natural compounds including control drug celecoxib interaction with COX-2. Data obtained from PyRx tool

**Ligand**	**Binding Affinity**	**rmsd/ub**	**rmsd/lb**
**5f1a_Arbortristoside-E**	-10.1	0	0
**5f1a_beta-Sitosterol**	-9.6	0	0
**5f1a_astragalin**	-9.2	0	0
**5f1a_beta-Amyrin**	-9.1	0	0
**5f1a_arborside-A**	-9.1	0	0
**5f1a_Arborside-C**	-9.1	0	0
**5f1a_Quercetin**	-9	0	0
**5f1a_DB00482 (Control Drug Celecoxib)**	-8.8	0	0
**5f1a_Arborside-B**	-8.8	0	0
**5f1a_nicotiflorin**	-8.6	0	0
**5f1a_lupeol**	-8.6	0	0
**5f1a_Nyctanthic_acid**	-8.6	0	0
**5f1a_Apigenin**	-8.5	0	0
**5f1a_Kaemferol**	-8	0	0
**5f1a_Nyctanthoside**	-7.9	0	0
**5f1a_Arbortristoside-C**	-7.8	0	0
**5f1a_Arbortristoside-B**	-7.7	0	0
**5f1a_6beta-Hydroxyloganin**	-7.4	0	0
**5f1a_Alpha-xylopyranoside**	-7.3	0	0
**5f1a_hentriacontane**	-6.5	0	0
**5f1a_p-cymene**	-6.4	0	0

**5f1a_methyl_salicylate**	-6.2	0	0
**5f1a_benzoic_acid**	-6.1	0	0
**5f1a_alpha-Pinene**	-5.5	0	0
**5f1a_Mannitol**	-5.2	0	0
**5f1a_1-deconol**	-5.1	0	0
**5f1a_arbortristoside-D**	-1.9	0	0

Table S3: ADME prediction from SwissADME (GI=Gastro intestinal, BBB=Blood Brain Barrier, Pgp=P glycoprotein, CYP=Cytochrome, log Kp= skin permeation)

**Compounds**	**GI absorption**	**BBB permeant**	**Pgp substrate**	**CYP1A2 inhibitor**	**CYP2C19 inhibitor**	**CYP2C9 inhibitor**	**CYP2D6 inhibitor**	**CYP3A4 inhibitor**	**log Kp (cm/s)**
									negative the log K_p the less skin permeant the molecule
**Control (Celecoxib)**	High	No	No	Yes	No	Yes	No	No	-6.21
**Arb_E**	Low	No	No	No	No	No	No	No	-9.95
**Beta-Sito**	Low	No	No	No	No	No	No	No	-2.2

Table S4. Drug-likeness prediction from SwissADME server (MW=Molecular Weight, TPSA= total polar surface area, Consensus Log P= average of all predicted Log Po/w													
**Compounds**	**MW (g/mol)**	**Rotatable bonds**	**H-bond acceptors**	**H-bond donors**	**TPSA (Å²)**	**Consensus Log P**	**Lipinski violations**	**Ghose violations**	**Veber violations**	**Egan violations**	**Muegge violations**	**Bioavailability Score**	**Synthetic Accessibility**
	MW between 150 and 500 g/mol	no more than 9 rotatable bonds			TPSA between 20 and 130 Å^2	not higher than 6						not less than 0.25	normalized between 1 (easy synthesis) and 10 (very difficult synthesis)
**Control (Celecoxib)**	381.37	4	7	1	86.36	3.4	0	1	0	0	0	0.55	2.74
**Arb_E**	566.55	10	13	5	190.67	-0.14	2	4	1	1	2	0.11	6.51
**Beta-Sito**	414.71	6	1	1	20.23	7.19	1	3	0	1	2	0.55	6.3

Table S5: toxicity prediction. Data obtained from pkCSM server

	AMES toxicity	Max. tolerated dose (Human)	hERG I inhibitor	hERG II inhibitor	Oral Rat Acute Toxicity (LD50)	Oral Rat Chronic Toxicity (LOAEL)	Hepatotoxicity	Skin sensitisation	T. pyriformis toxicity	Minnow toxicity
**Standard cut-off**	**Positive mutagenic**	**<= 0.477 log(mg/kg/day)**				**lowest**			**> -0.5 log ug/L**	**Log LC50< -0.3**
**Compounds**										
**Control (Celecoxib)**	Yes	0.021	No	No	2.027	0.963	No	No	0.527	0.86
**Arb_E**	No	-0.151	No	Yes	3.197	3.25	No	No	0.285	5.539
**Beta-Sito**	No	-0.621	No	Yes	2.552	0.855	No	No	0.43	-1.802

Figure S1: List of best docked poses of all selected receptors and their interaction with **Arbortristoside-E**

Enzymes	**Arbortristoside-E**
COX-1<br><br>(A)	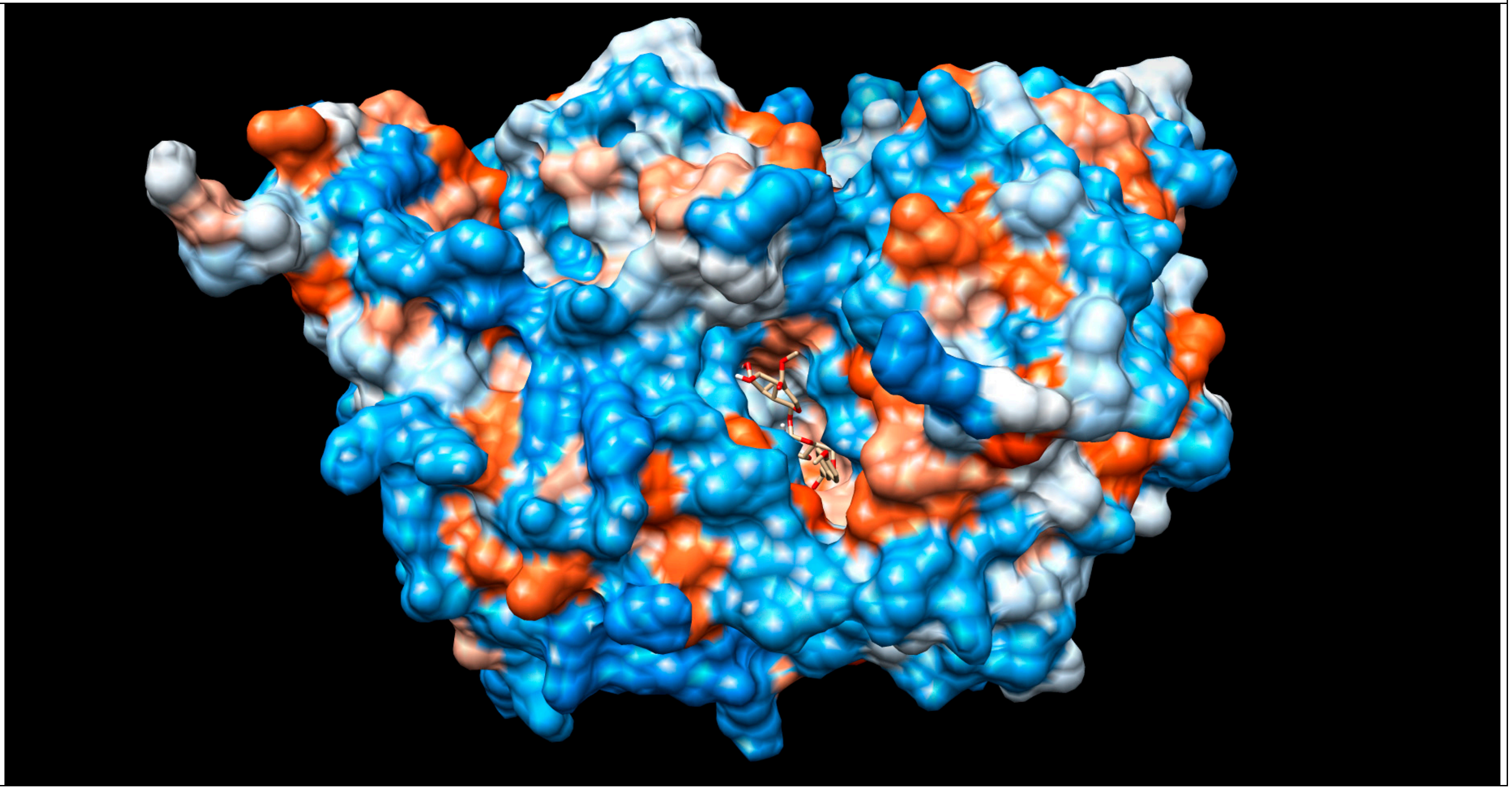

COX-2<br><br>(B)	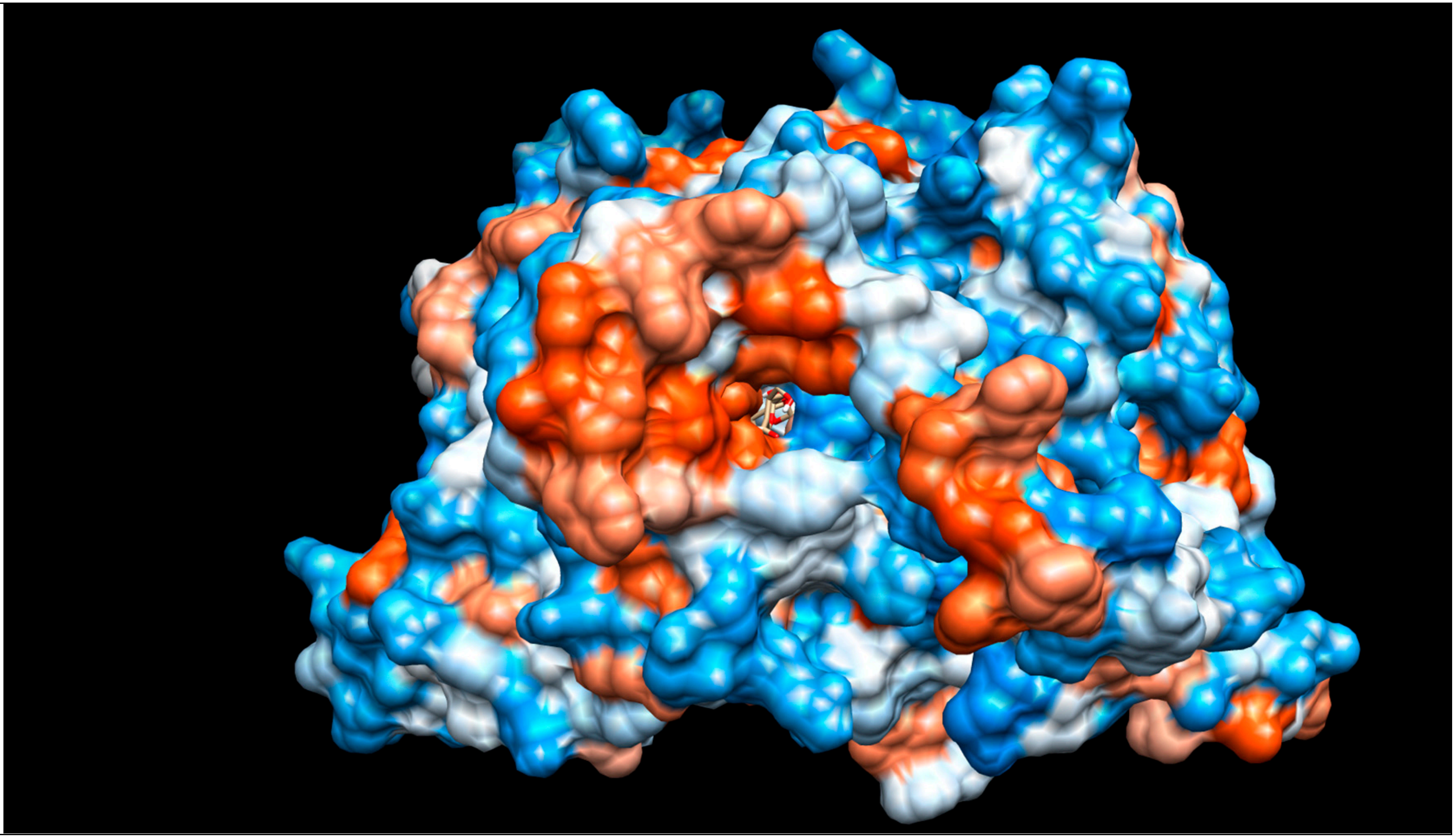

PDE4<br><br>(C)		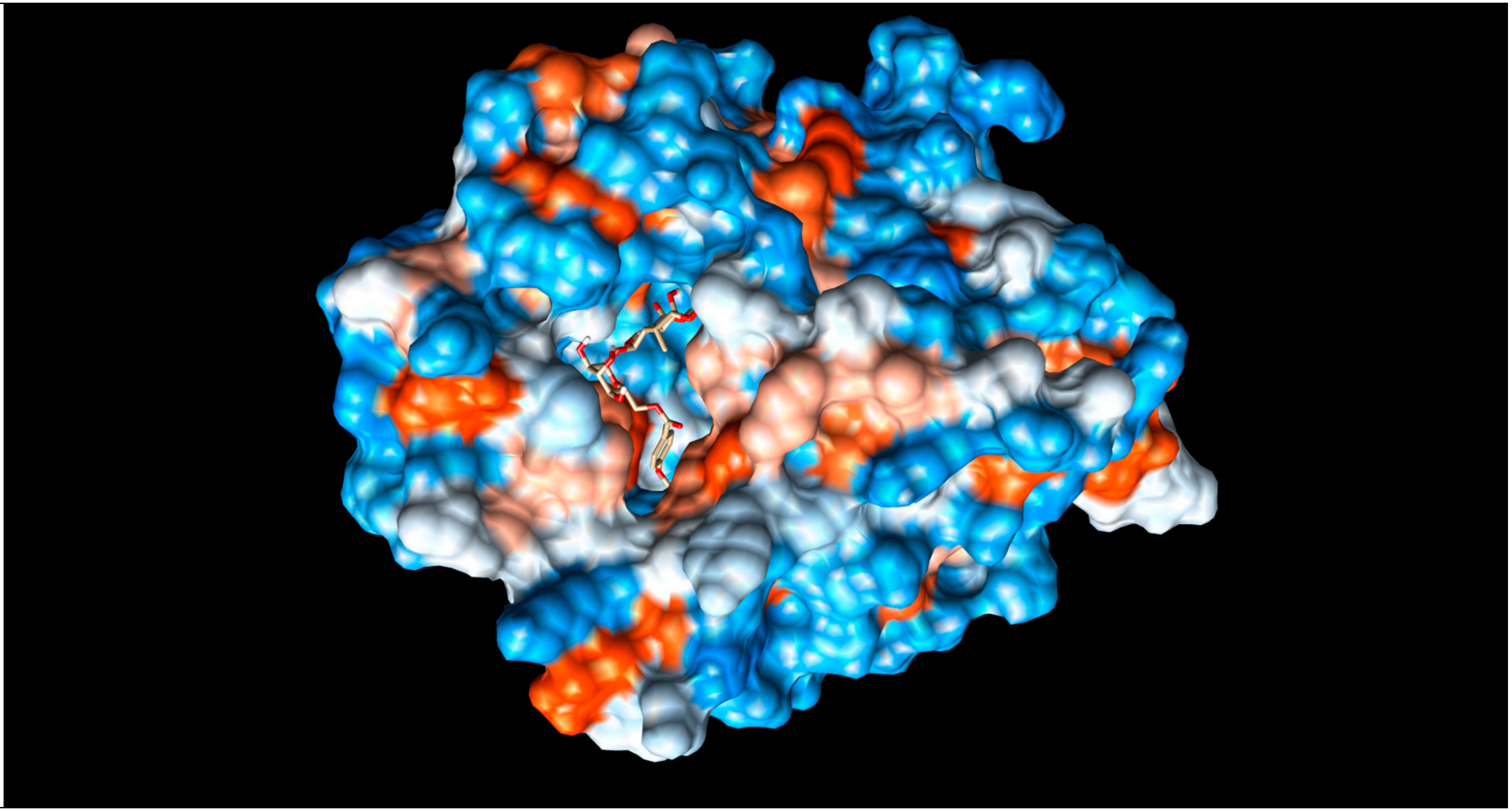

PDE7<br><br>(D)		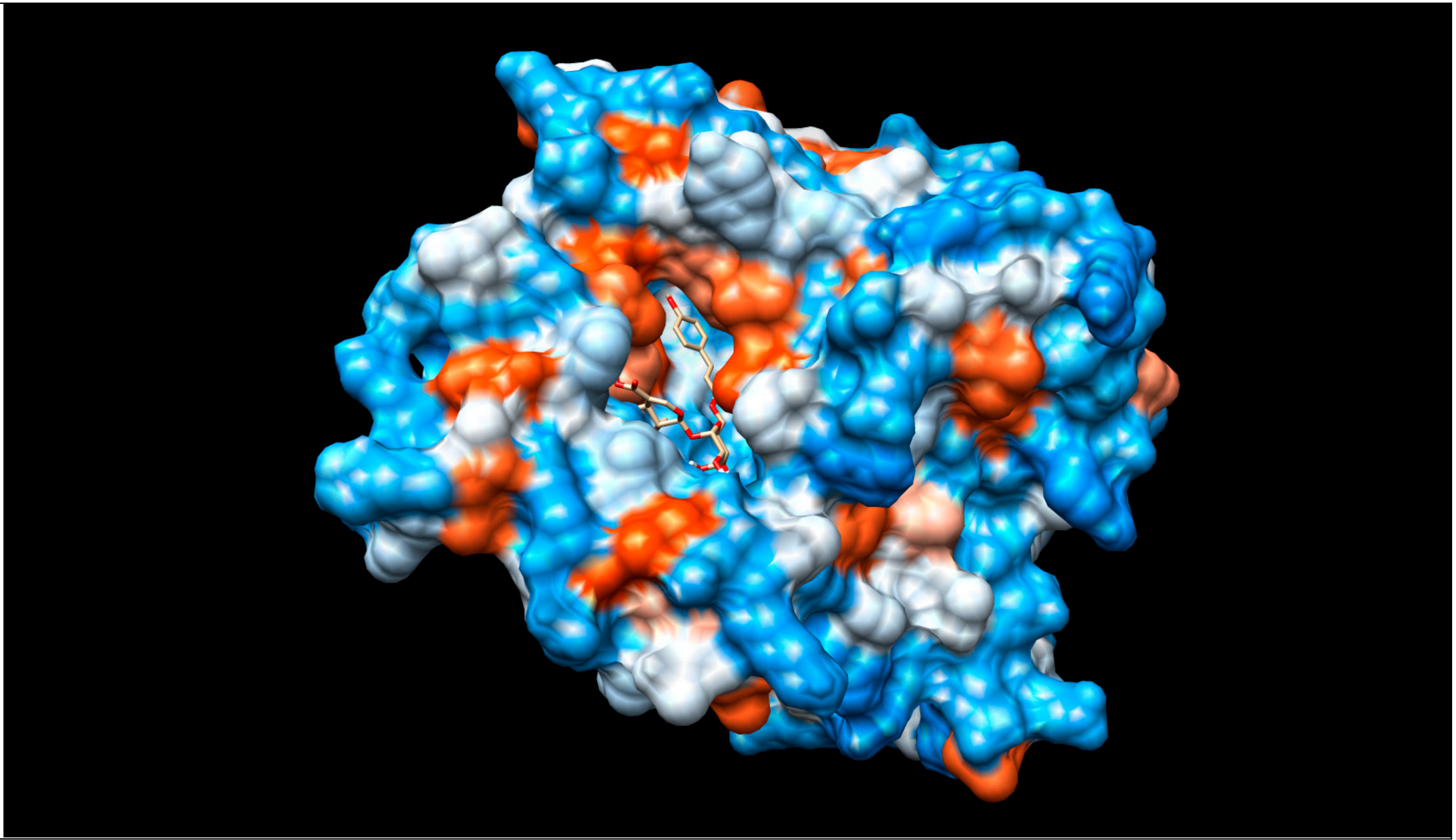

IL-17A<br><br>(E)	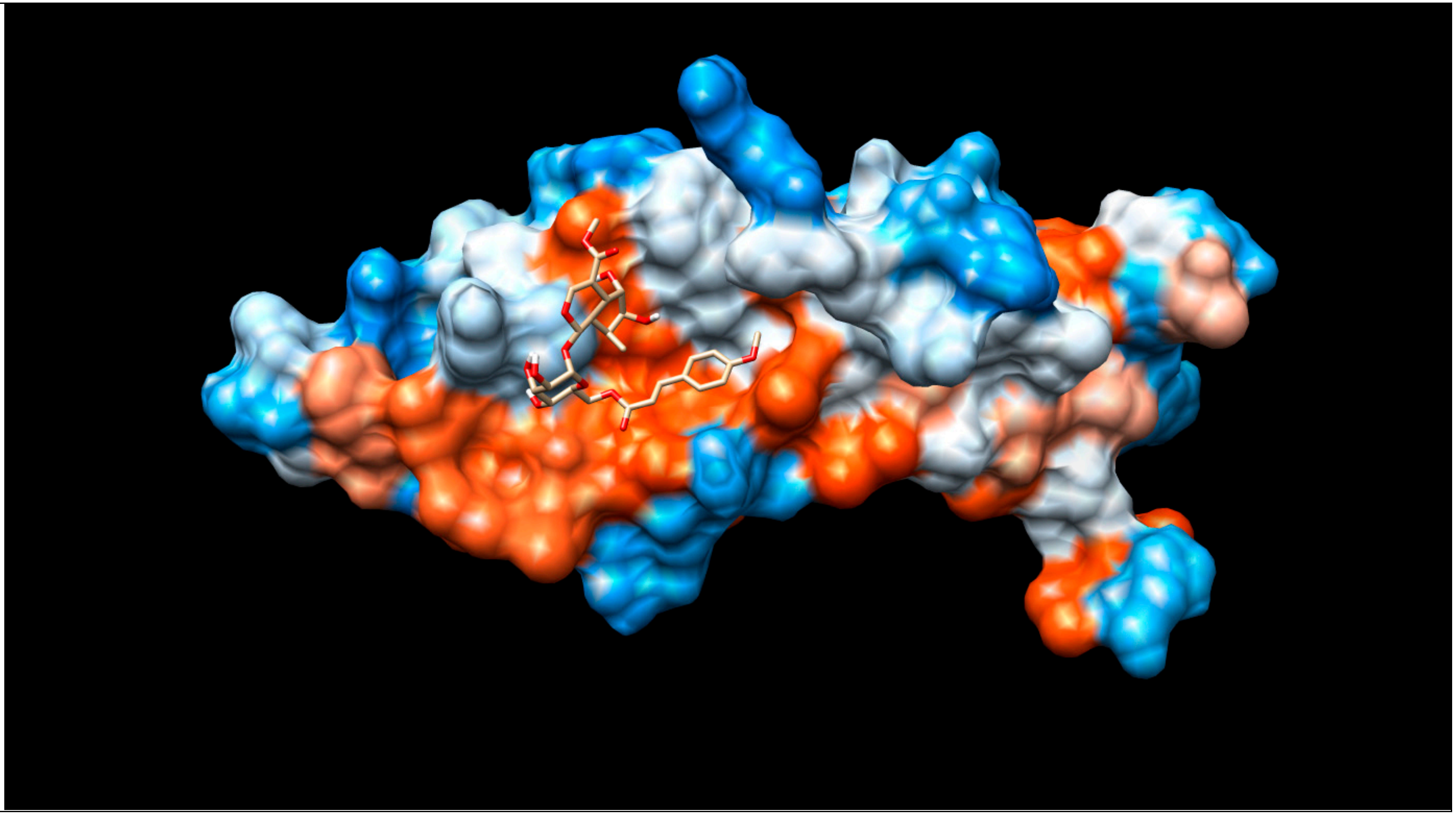

IL-17D<br><br>(F)	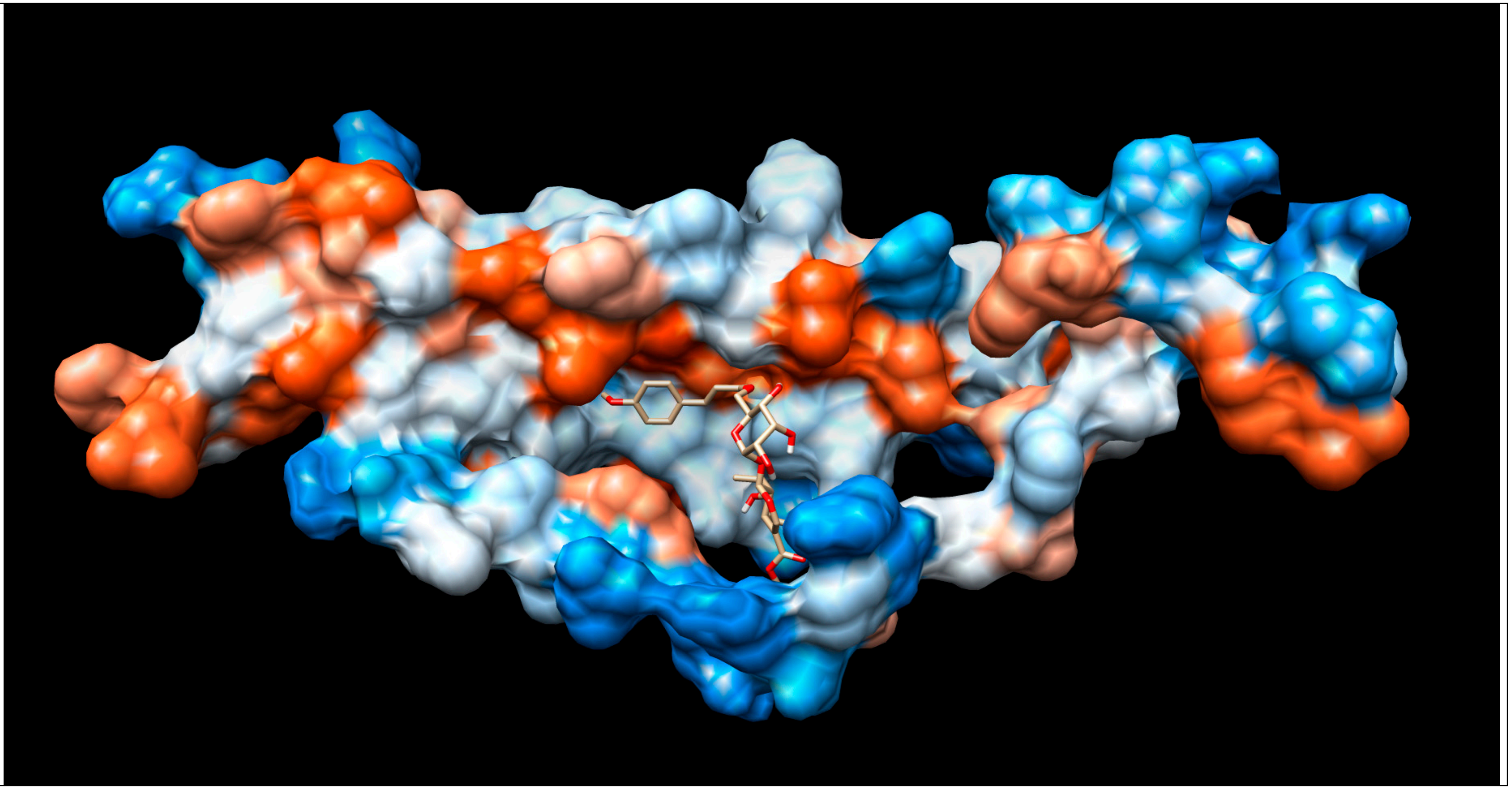

TNF-α<br><br>(G)	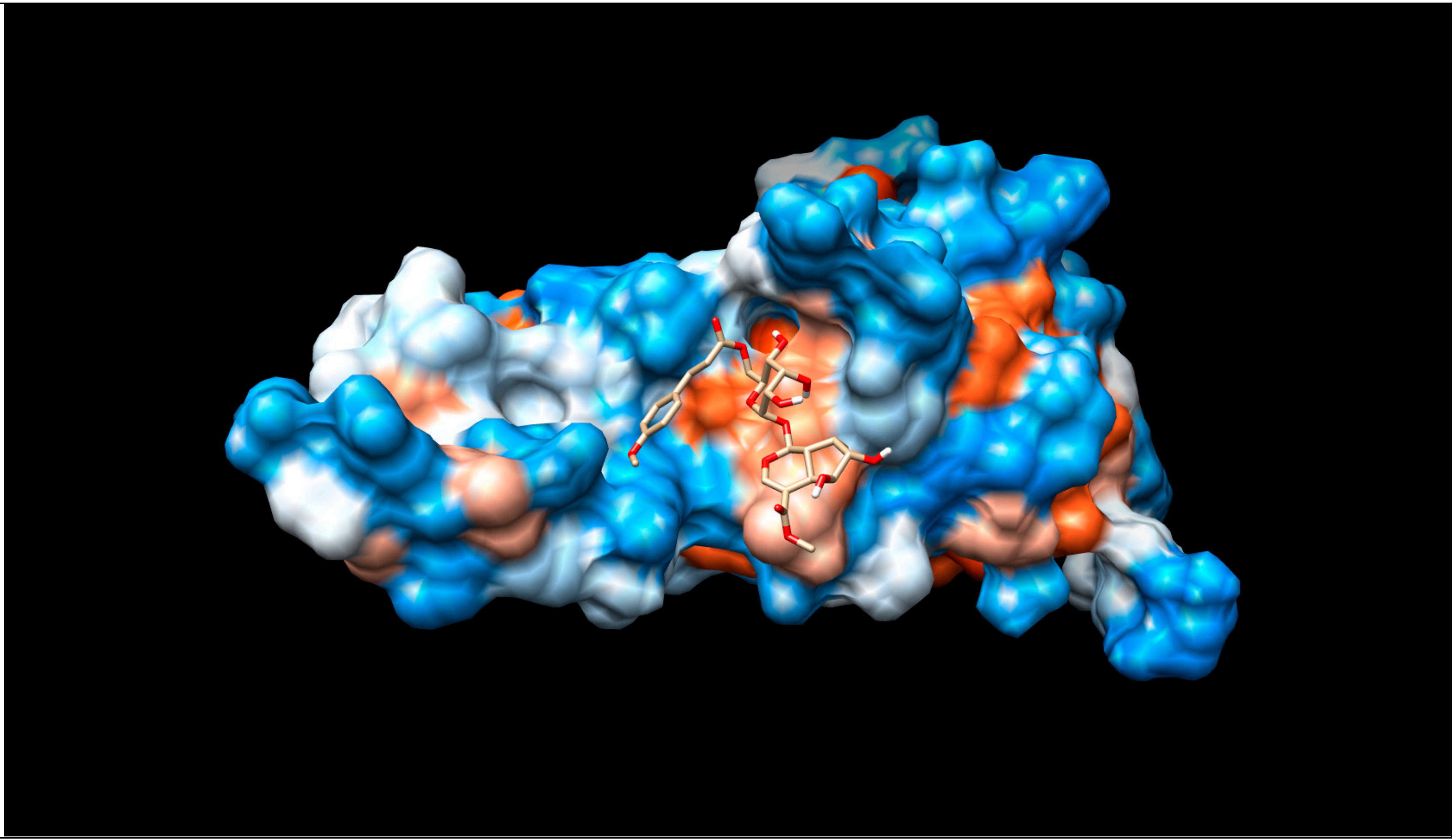

IL-1β<br><br>(H)	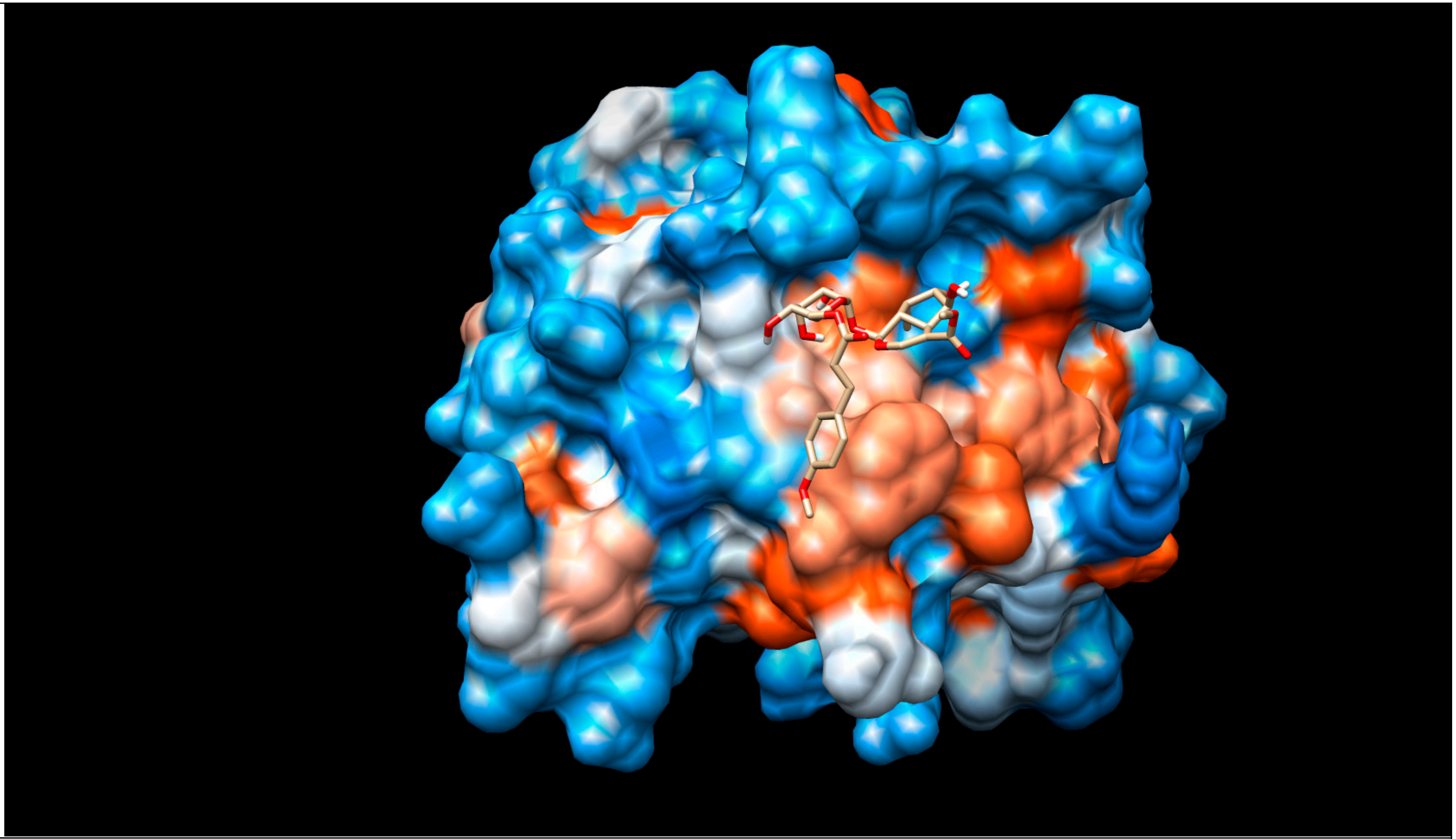

Prostagla ndin E2<br><br>(I)	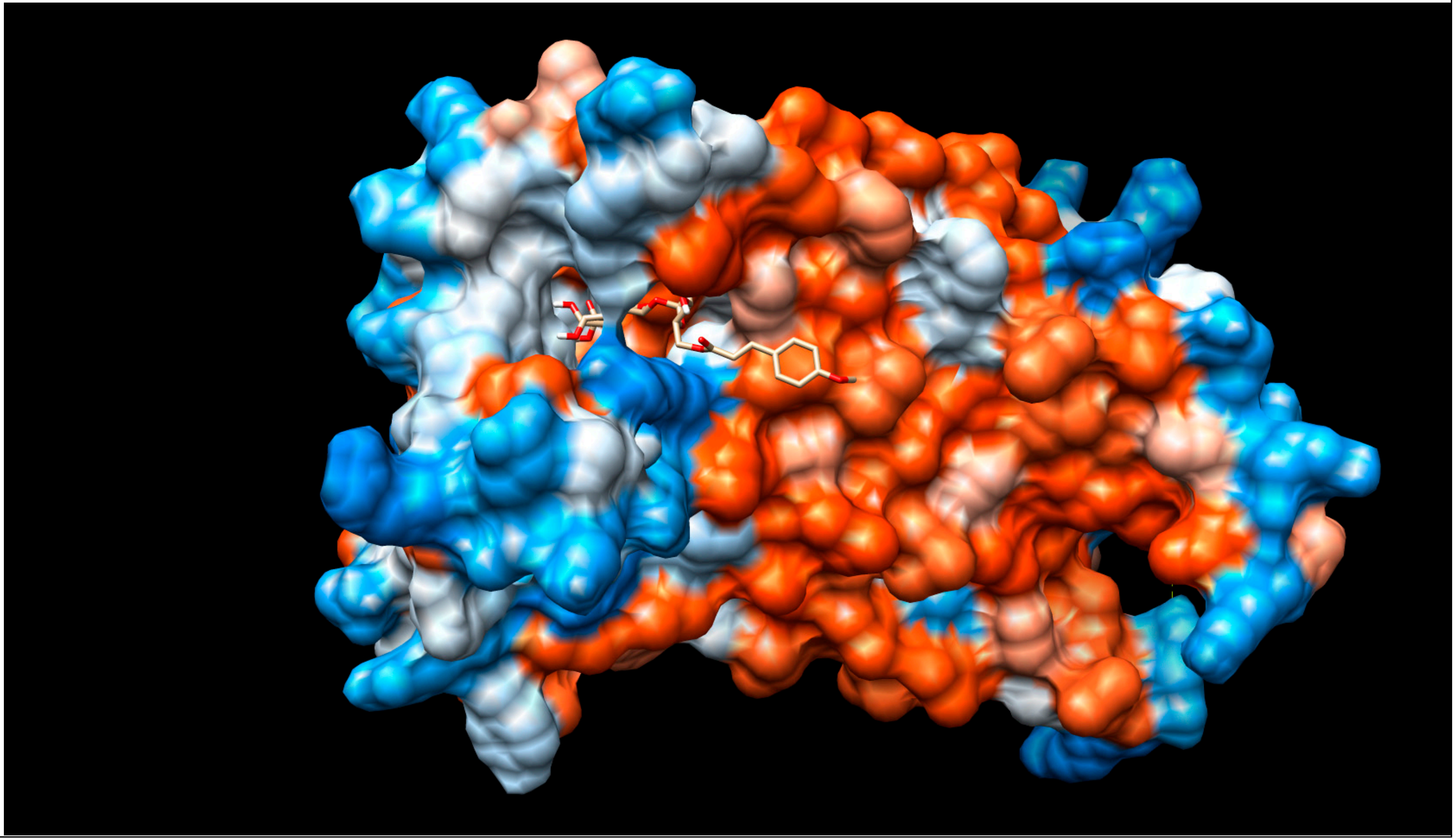

PGF<br><br>(J)	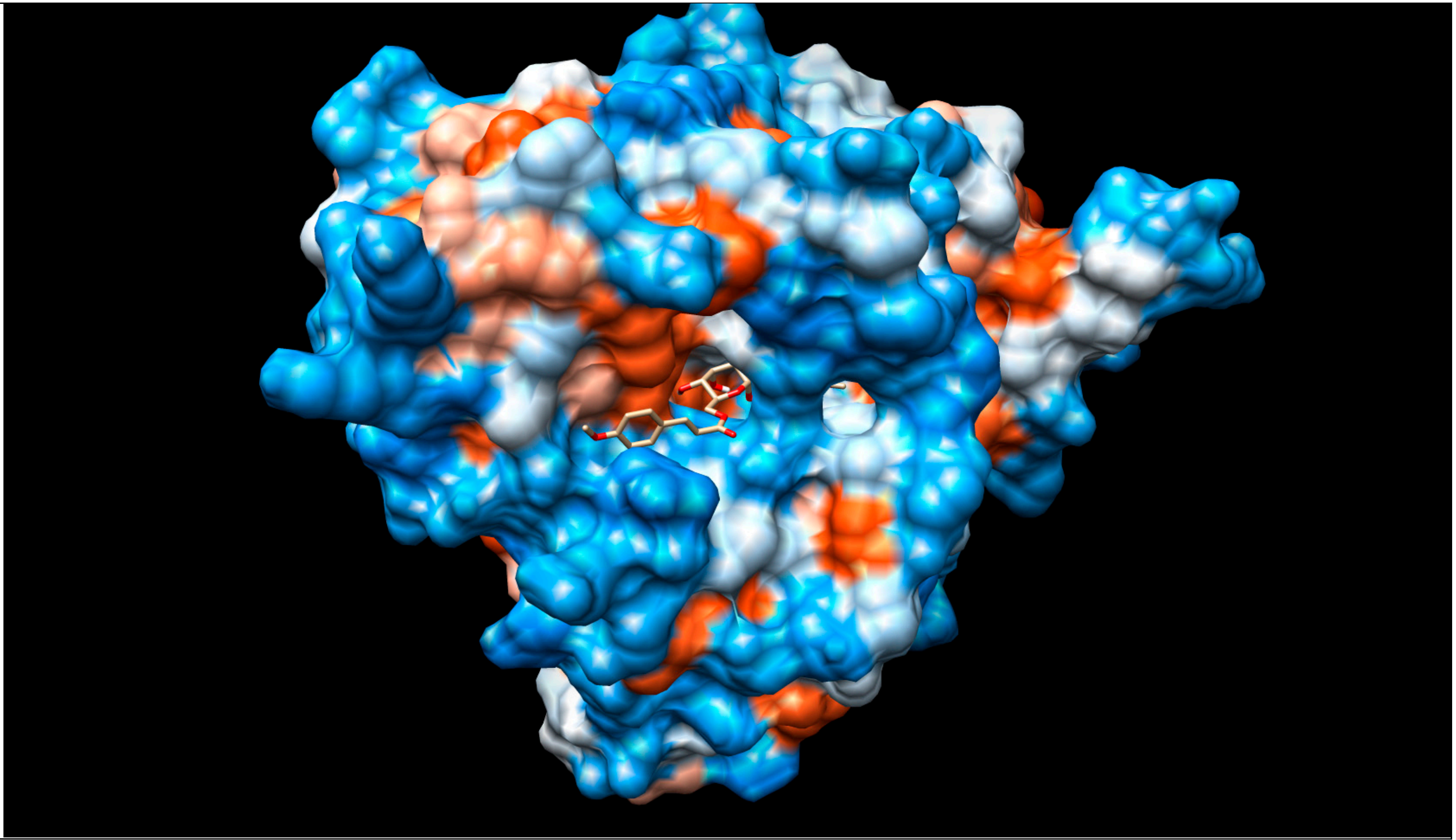

Figure S2: List of best-docked poses of all selected receptors and their interaction with Beta-sitosterol

Enzymes	**Beta-sitosterol**
COX-1<br><br>(A)	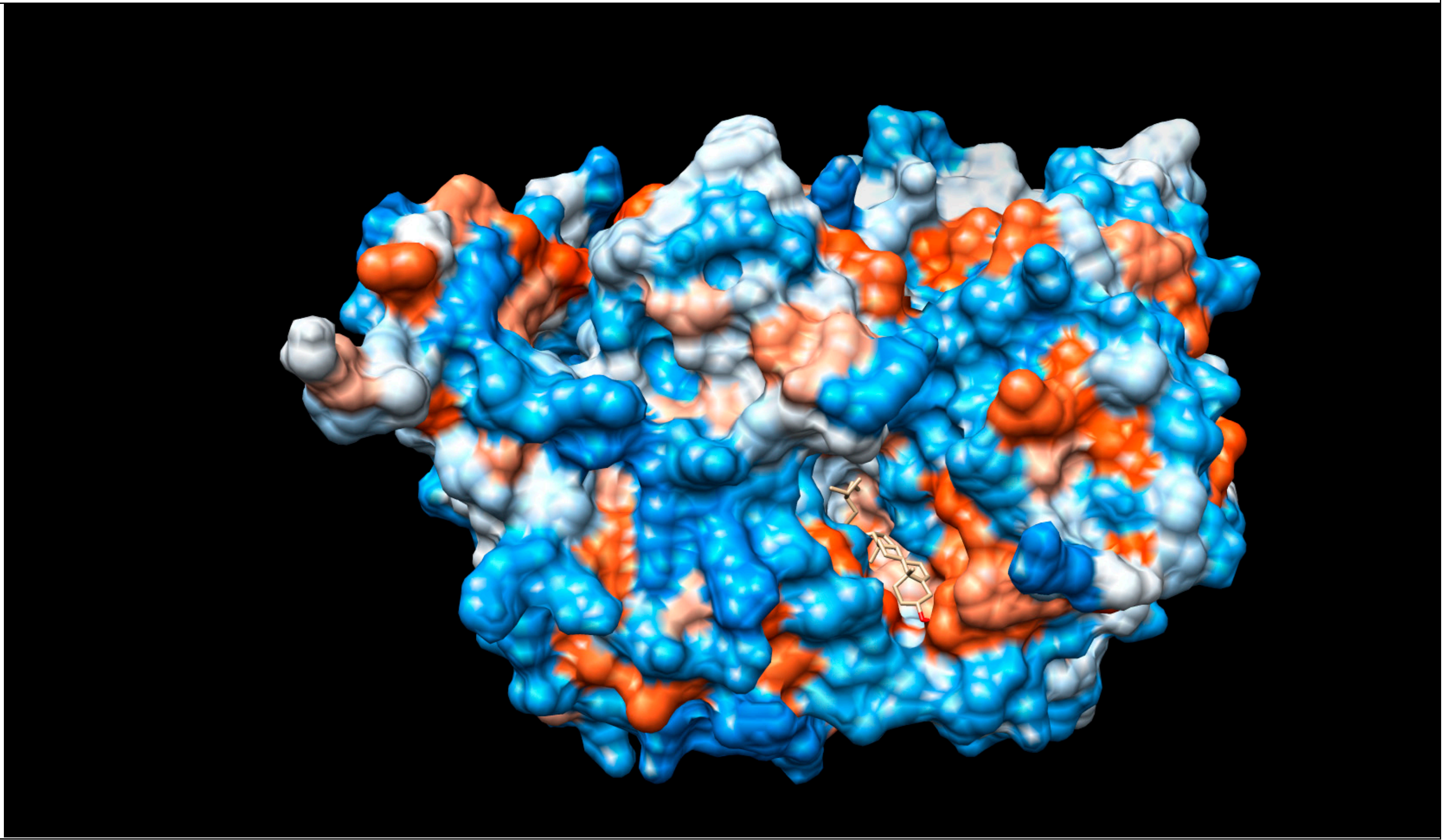

COX-2<br><br>(B)	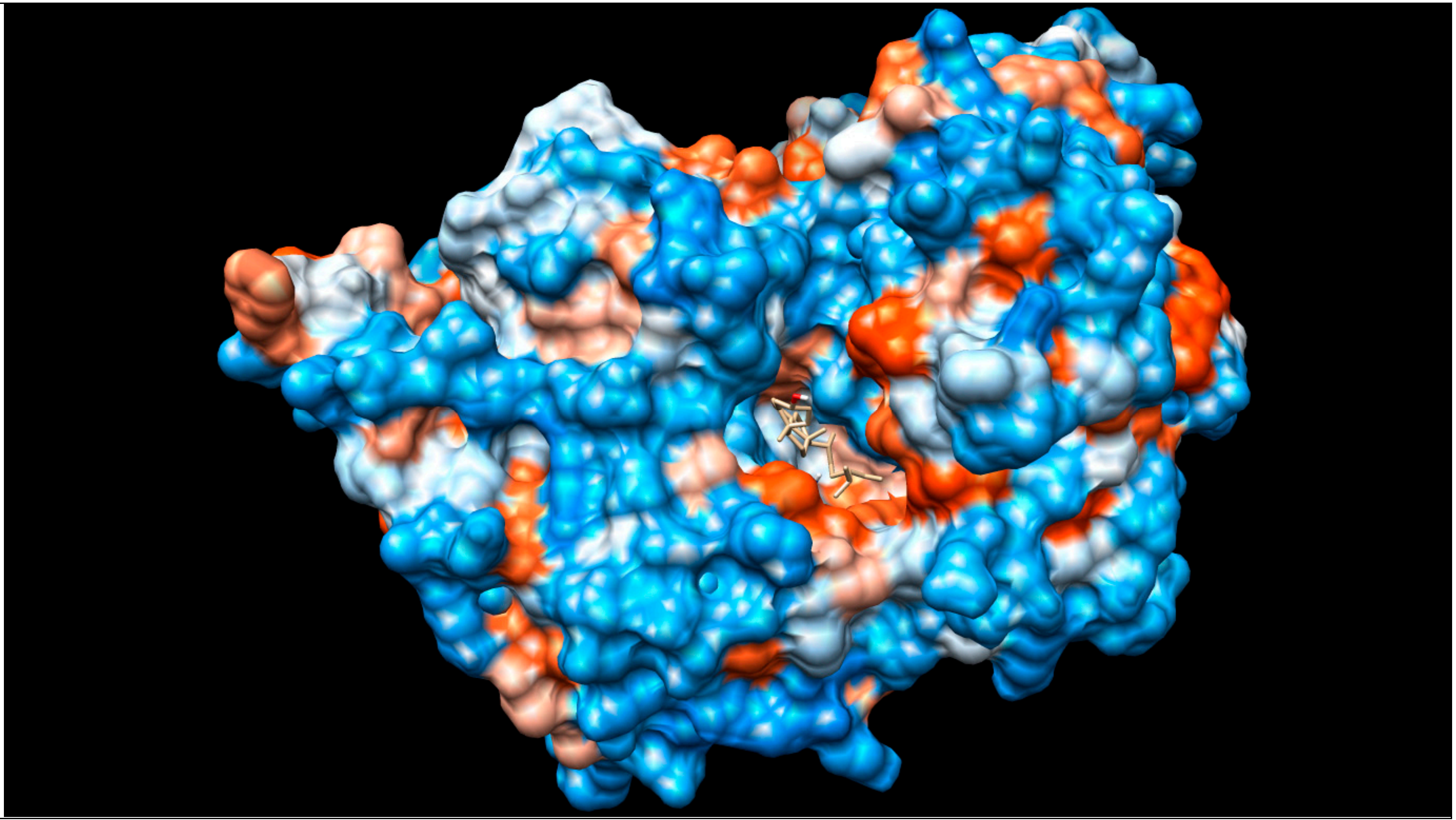

PDE4<br><br>(C)	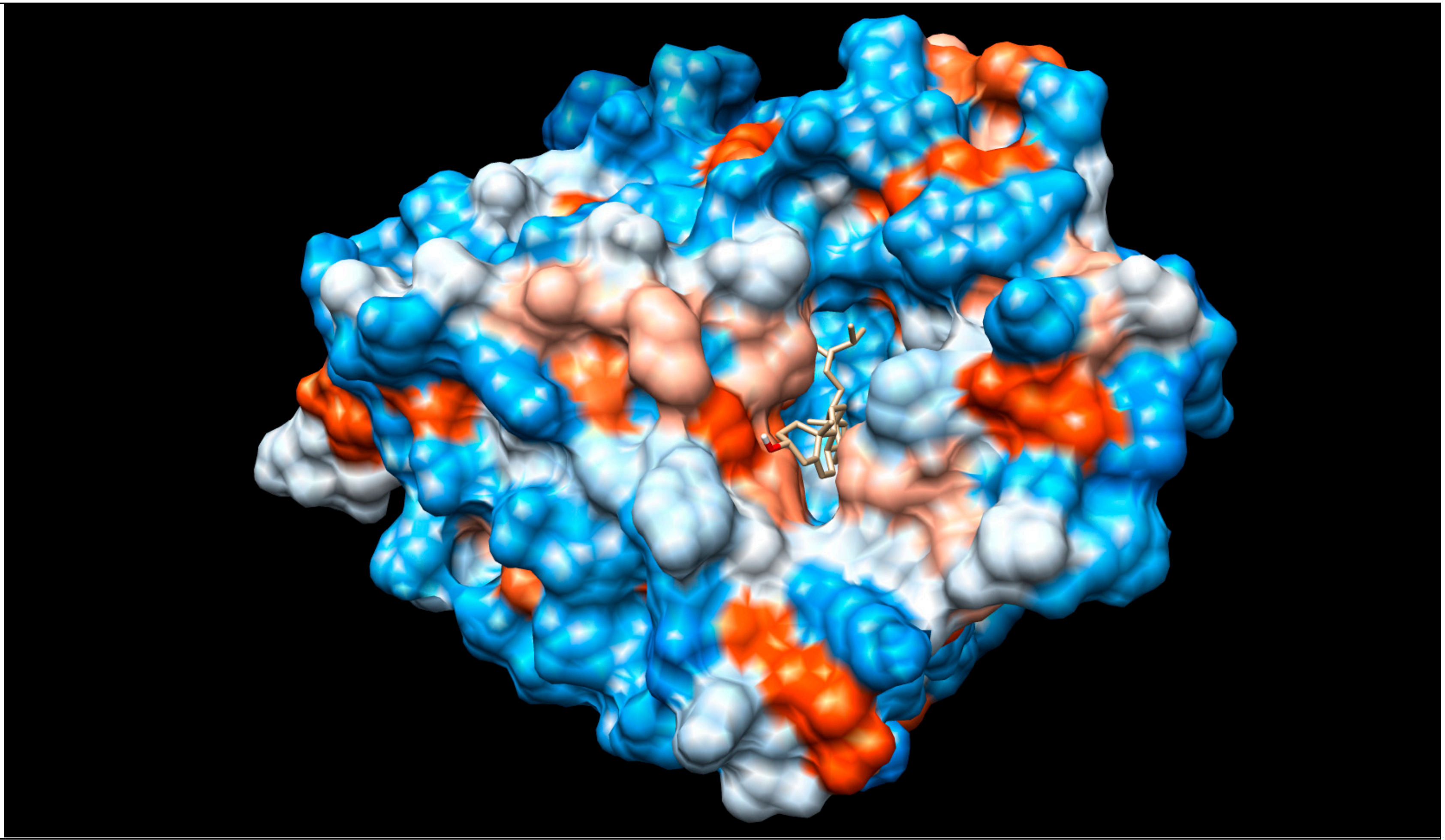

PDE7<br><br>(D)	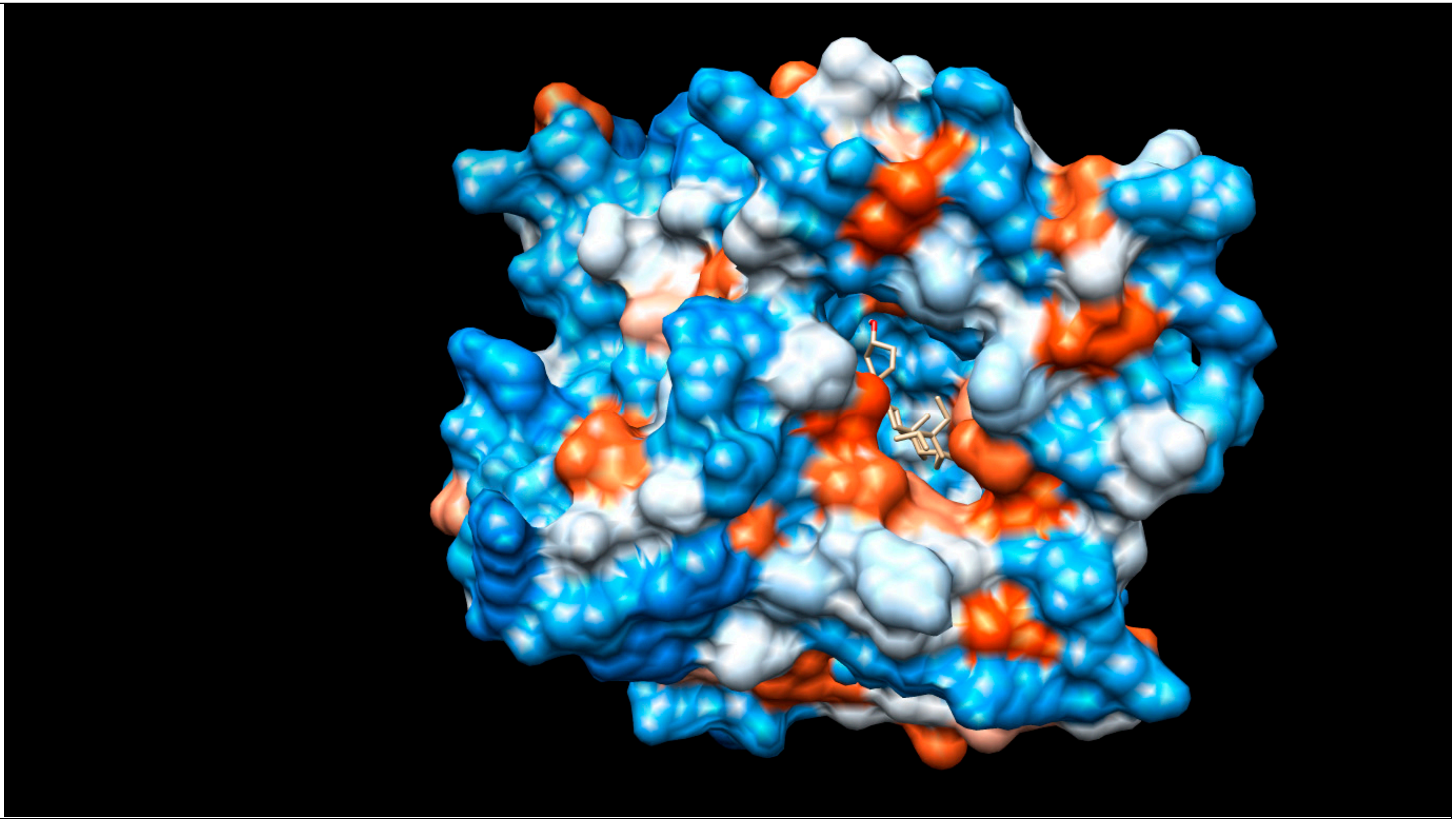

IL-17A<br><br>(E)	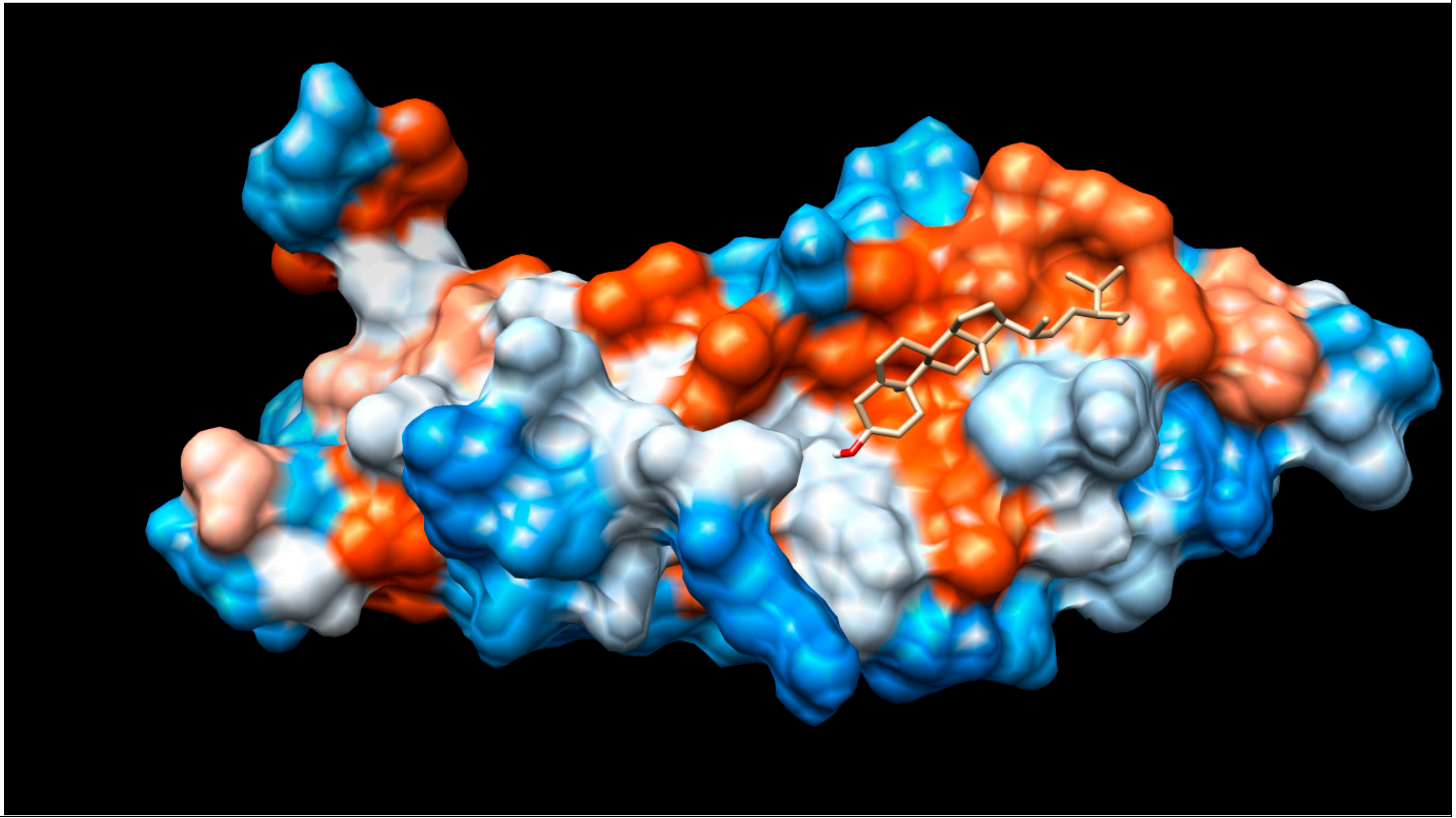

IL-17D<br><br>(F)	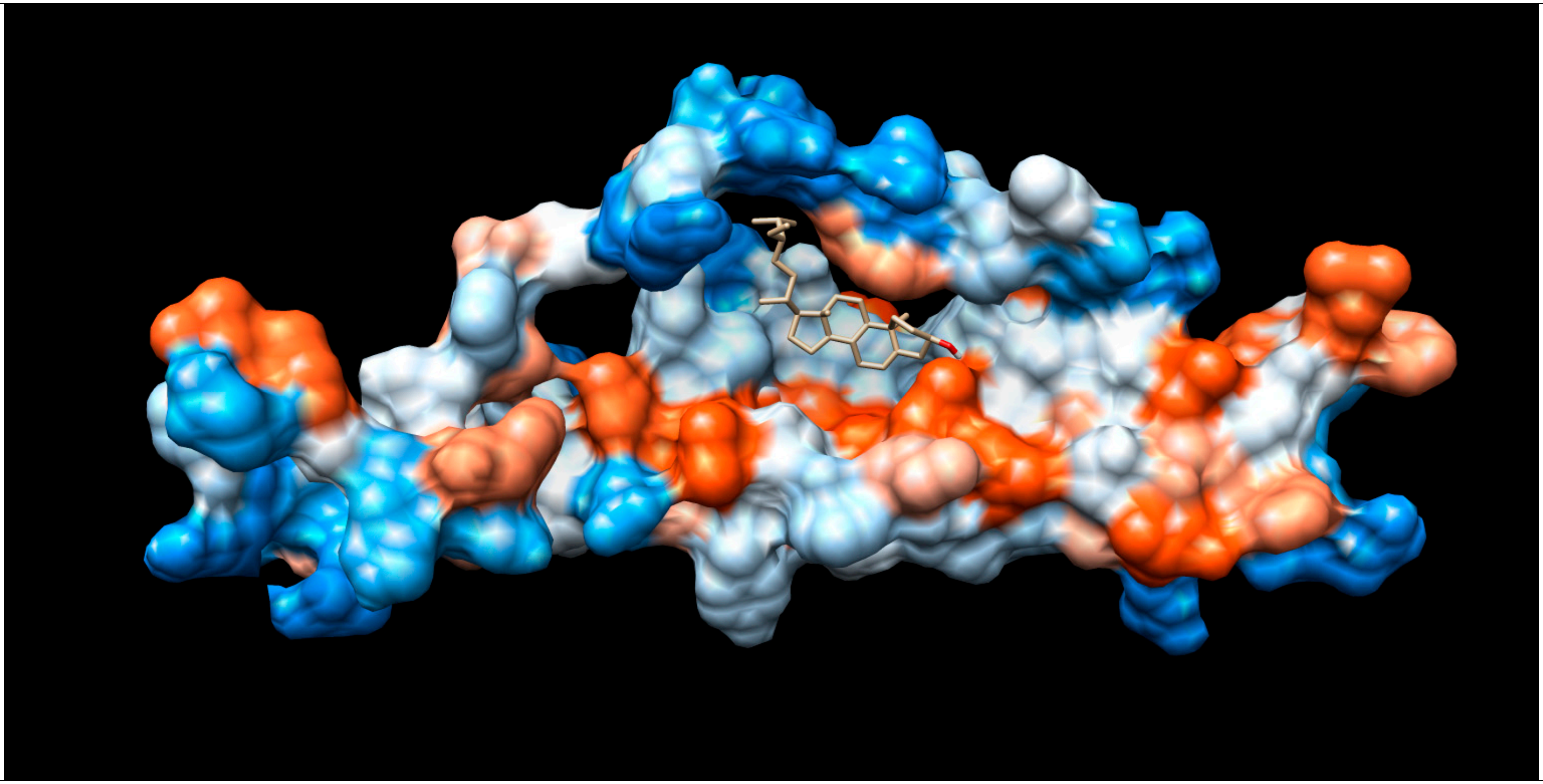

TNF-α<br><br>(G)	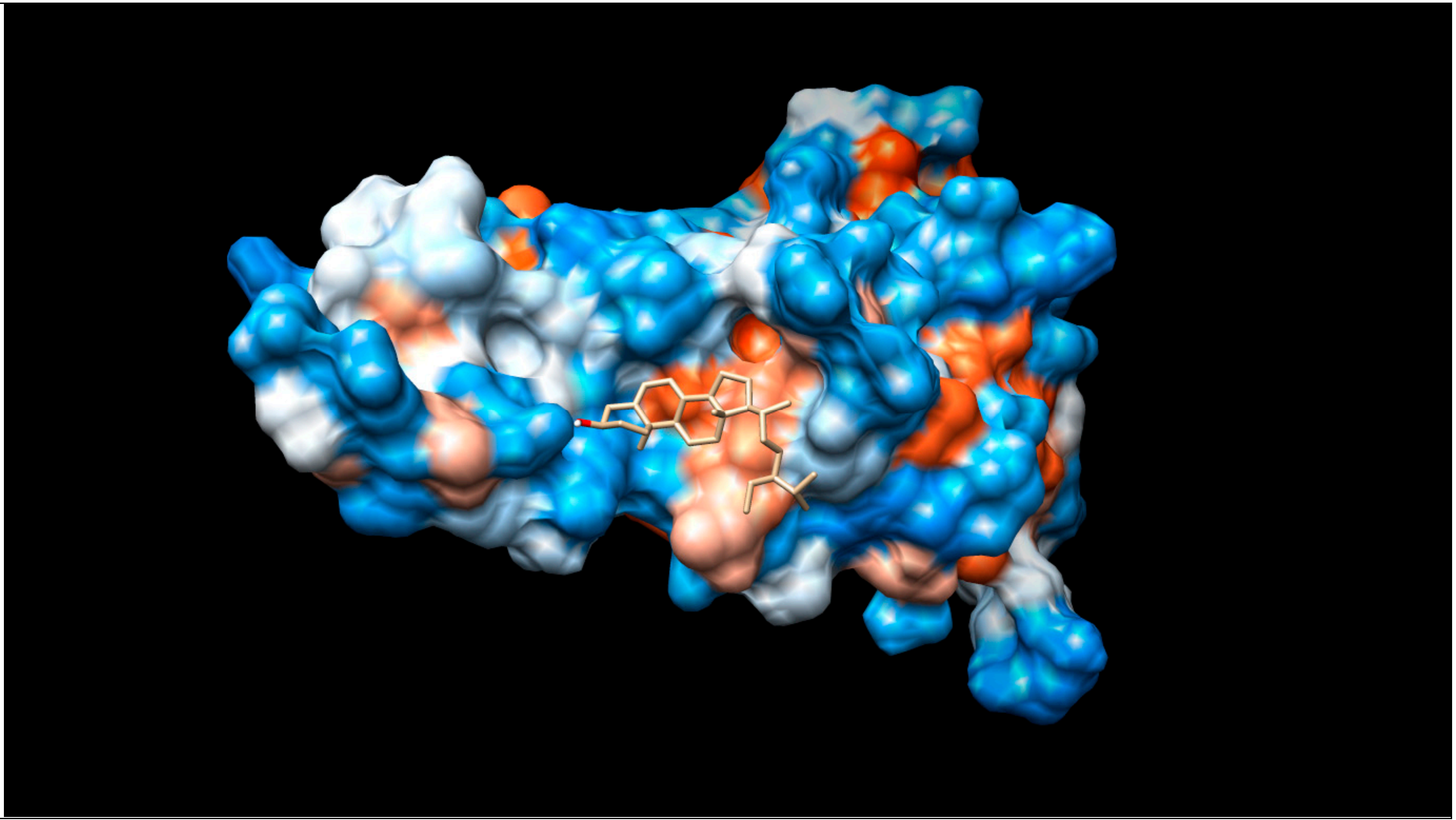

IL-1β<br><br>(H)	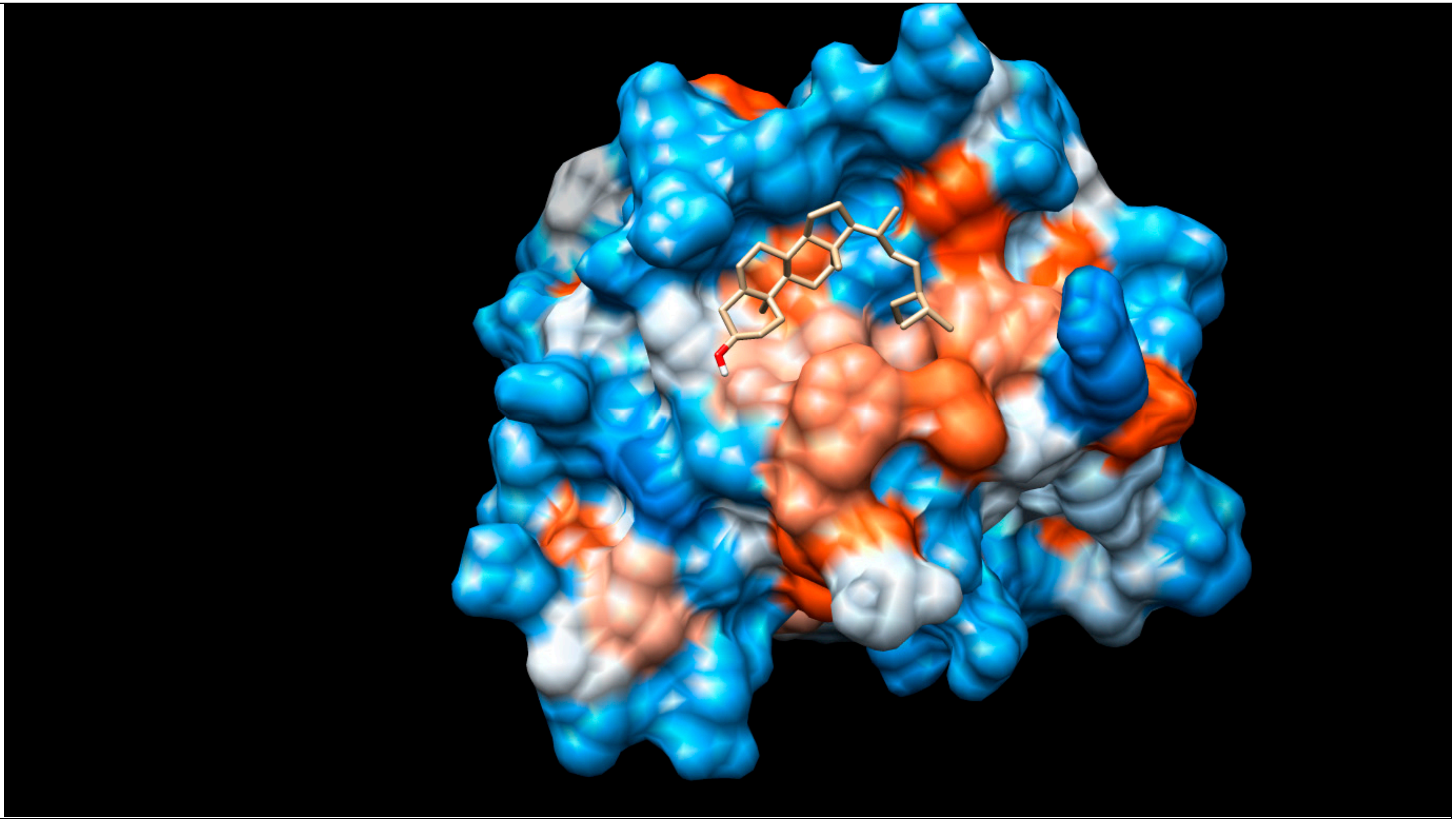

Prostaglandin E2<br><br>(I)	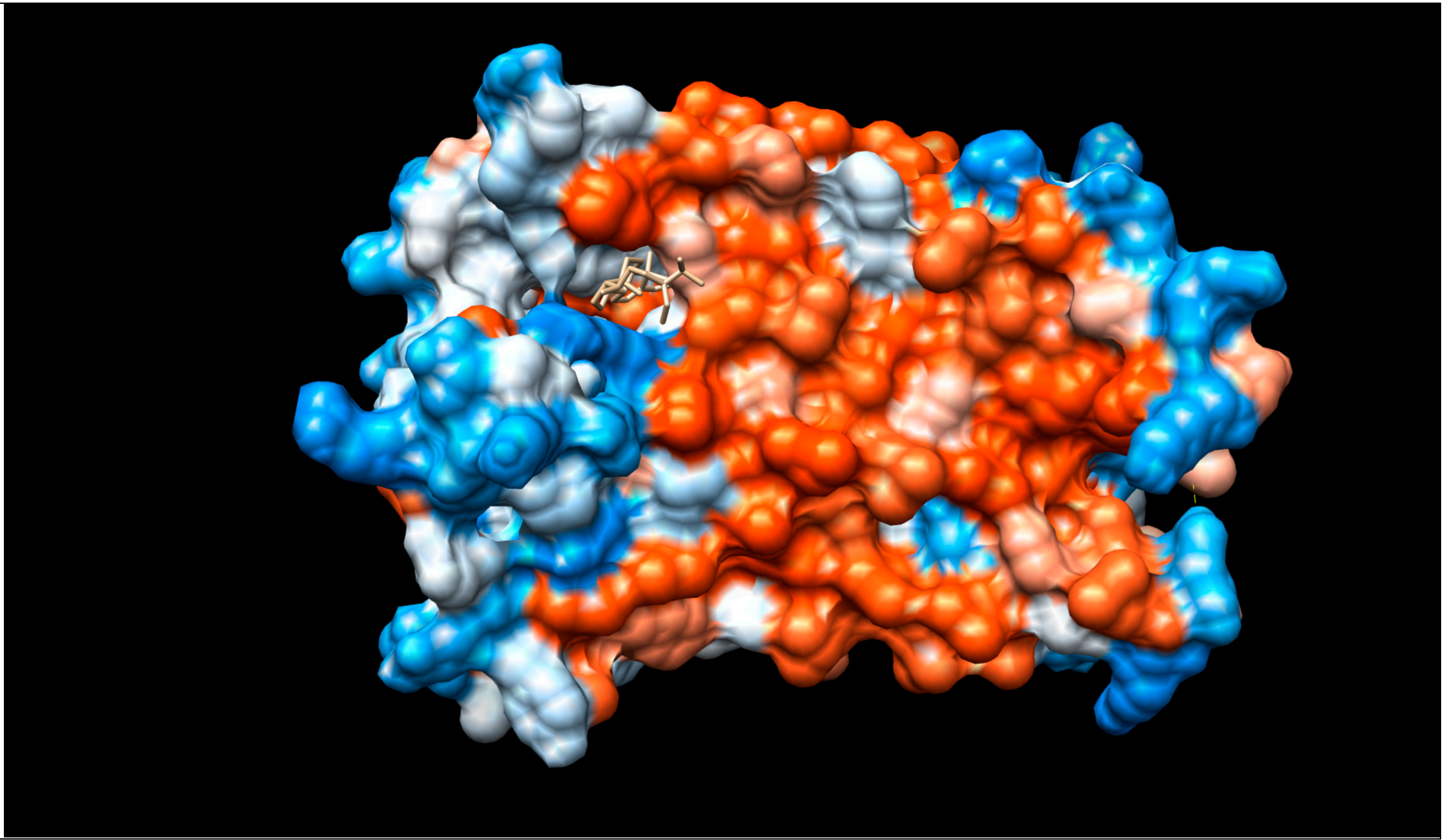

PGF<br><br>(J)	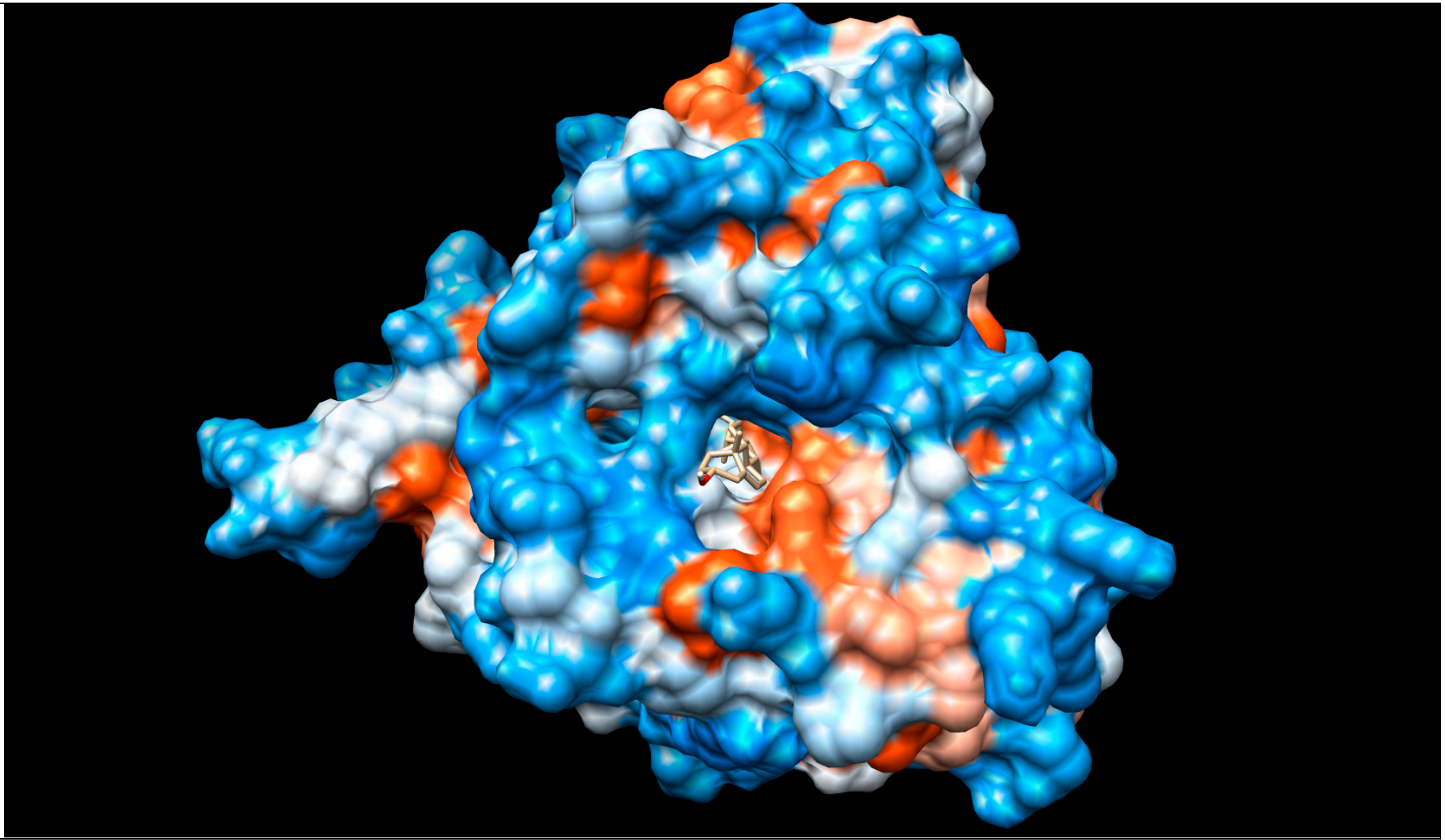
